# New Thermophilic α/β Class Epoxide Hydrolases Found in Metagenomes From Hot Environments

**DOI:** 10.3389/fbioe.2018.00144

**Published:** 2018-10-16

**Authors:** Erica Elisa Ferrandi, Christopher Sayer, Simone Antonio De Rose, Elisa Guazzelli, Carlotta Marchesi, Vahid Saneei, Michail N. Isupov, Jennifer A. Littlechild, Daniela Monti

**Affiliations:** ^1^Istituto di Chimica del Riconoscimento Molecolare, C.N.R., Milan, Italy; ^2^The Henry Wellcome Building for Biocatalysis, Biosciences, College of Life and Environmental Sciences, University of Exeter, Exeter, United Kingdom

**Keywords:** epoxide hydrolase, metagenomics, industrial biocatalysis, stereoselectivity, protein structure

## Abstract

Two novel epoxide hydrolases (EHs), Sibe-EH and CH65-EH, were identified in the metagenomes of samples collected in hot springs in Russia and China, respectively. The two α/β hydrolase superfamily fold enzymes were cloned, over-expressed in *Escherichia coli*, purified and characterized. The new EHs were active toward a broad range of substrates, and in particular, Sibe-EH was excellent in the desymmetrization of *cis*-2,3-epoxybutane producing the (2*R*,3*R*)-diol product with *ee* exceeding 99%. Interestingly these enzymes also hydrolyse (4*R*)-limonene-1,2-epoxide with Sibe-EH being specific for the *trans* isomer. The Sibe-EH is a monomer in solution whereas the CH65-EH is a dimer. Both enzymes showed high melting temperatures with the CH65-EH being the highest at 85°C retaining 80% of its initial activity after 3 h thermal treatment at 70°C making it the most thermal tolerant wild type epoxide hydrolase described. The Sibe-EH and CH65-EH have been crystallized and their structures determined to high resolution, 1.6 and 1.4 Å, respectively. The CH65-EH enzyme forms a dimer via its cap domains with different relative orientation of the monomers compared to previously described EHs. The entrance to the active site cavity is located in a different position in CH65-EH and Sibe-EH in relation to other known bacterial and mammalian EHs.

## Introduction

Epoxide hydrolases (EHs, EC 3.3.2.9) are enzymes that catalyze the *in vivo* hydrolysis of an epoxide ring to the corresponding vicinal diols. This activity was originally reported in mammalian cells more than 40 years ago (Jerina et al., 1968; Brooks et al., 1970). Since then, EHs have been found in a wide range of different organisms including bacteria and fungi (Arand et al., 1999; Kotik et al., 2007; Liu et al., 2007), plants and insects (Kiyosue et al., 1994; Stapleton et al., 1994; Guo et al., 1998; Linderman et al., 2000; Morisseau et al., 2000). The physiological roles of EHs have been widely studied. These enzymes have been shown to be involved in the detoxification of xenobiotics in mammals (Oesch, 1973; Morisseau and Hammock, 2005) and fungi (Sutherland, 1992), in the general defense system against pathogens and stress response in plants (Kiyosue et al., 1994; Guo et al., 1998) and in hormone biosynthesis in insects (Linderman et al., 2000).

The majority of EHs described to date belong to the α/β hydrolase superfamily, where the core domain with a particular direction and connectivity of β-strands is covered by a mainly α-helical cap domain, with the active site located on the interface of the two domains (Widersten et al., 2010). An Asp-His-Asp/Glu catalytic triad located in the core domain is essential for the epoxide hydrolytic activity. The reaction proceeds via a two-steps catalytic mechanism involving the formation of a covalently bound enzyme-substrate intermediate. The first step of the reaction is the nucleophilic attack by the catalytic aspartate carboxylate group on one of the epoxide carbons of the substrate, resulting in the formation of an ester intermediate. The subsequent nucleophilic attack on the ester intermediate by an activated water molecule results in the formation of a diol product. Two conserved tyrosine residues in the cap domain point toward the catalytic triad and participate in the catalytic mechanism by binding the substrate and activating its protonation for catalysis (Nardini et al., 1999).

In recent years EHs have found industrial applications where they are used for the synthesis of optically pure chiral epoxides and 1,2-diols, both by the kinetic resolution of racemic epoxide mixtures, as well as through enantioconvergent processes or by the de-symmetrisation of *meso*-epoxides. The products are valuable building blocks for the synthesis of different pharmaceutical and agrochemical bioactive molecules (Bala and Chimni, 2010; Kotik et al., 2012; Wohlgemuth, 2015; Archelas et al., 2016). For example, EHs have been employed for the preparation of optically active phenylpropylene oxides which are useful building blocks for the synthesis of antibiotics and C-glycosides (Capriati et al., 2004), (*R*)-*para*-nitrostyrene oxide, which is a precursor of the β-blocker Nifenalol (Pedragosa-Moreau et al., 1997), and (*S*)-*para*-chloro styrene oxide, a precursor of a *N*-methyl-D-aspartate receptors (NMDA) receptor antagonist (Karboune et al., 2005).

The biocatalytic application of EHs in the synthesis of enantiopure epoxides and 1,2-diols represents a green and efficient alternative to traditional chemical methods which usually involve the use of potentially toxic heavy metal-based catalysts with only low to moderate turnover frequencies (Katsuki and Sharpless, 1980; Kolb and Sharpless, 1992; Kolb et al., 1994; Jacobsen, 2000). The EHs offer some inherent advantages, such as a usually high regio- and stereoselectivity on different substrates and the lack of expensive cofactor requirements (Bala and Chimni, 2010).

However, the need of integrating a biocatalytic synthetic step into existing industrial processes frequently requires the exploitation of thermostable enzymes that do not demand the reaction mixture to be cooled and reheated between the synthetic chemistry and biocatalytic steps, thereby making the whole process cheaper and less time and energy consuming. In addition, thermostable biocatalysts are usually more stable under the process conditions such as the presence of organic solvents which are used to solubilize the substrates. The thermostable biocatalyst can also be used at ambient temperatures with a lower specific activity but for extended reaction times. It can be reused for further reaction cycles and easily recovered if it is immobilized (Vieille et al., 2001; Littlechild, 2015; Zarafeta et al., 2016).

New innovative approaches such as the use of metagenomics for the discovery of new enzymes with improved process performance and broader substrate specificity are being used. This will help to meet the growing demand for more efficient and stable biocatalysts that are more suitable for industrial applications (Singh et al., 2009; Wilson and Piel, 2013; Ferrer et al., 2016).

The metagenomics approach has recently been used to identify some novel enzymes with industrially interesting features (Zhao et al., 2004; Kotik et al., 2009, 2010; Ferrandi et al., 2015b; Berini et al., 2017). For example, BD-EHs, isolated from environmental samples by Zhao and co-workers (Diversa Corporation), that are able to catalyze the desymmetrization of bulky internal epoxides such as *cis*-stilbene oxide and its derivatives (Zhao et al., 2004). Another enzyme, Kau2-EH has been isolated from a bacterial biomass, which was extracted from a bio-filter installed in a sewage disposal plant. This enzyme has shown broad substrate specificity and has been successfully used for the kinetic resolution of racemic α,β-di-substituted aromatic epoxides and for the desymmetrization of *cis*-stilbene oxide resulting in nearly enantiomerically pure diol and epoxide products (Kotik et al., 2010; Zhao et al., 2015). New genes coding for putative EHs have been recently identified in metagenomes collected in the Andean forest soil and mangrove forest soil (Montaña et al., 2012; Jiménez et al., 2015). In our previous work, we have identified two new epoxide hydrolases belonging to the small subfamily of the limonene epoxide hydrolases (LEH, E.C. 3.3.2.8) in the metagenomes of samples collected in hot terrestrial environments (Ferrandi et al., 2015b). These novel LEHs, which differ from the α/β hydrolase fold EHs, have a smaller size, different fold and different catalytic mechanism (Barbirato et al., 1998). They show high thermostability with a melting temperature in excess of 70°C (Ferrandi et al., 2015b) and have been successfully used for the resolution of (+)- and (–)-*cis*/*trans*-limonene oxides at the gram scale (Ferrandi et al., 2015a).

Soluble α/β EHs from a number of mammalian (Argiriadi et al., 1999; Pilger et al., 2015), plant (Mowbray et al., 2006), fungal (Reetz et al., 2009), insect (Zhou et al., 2014) and bacterial (Nardini et al., 1999; Biswal et al., 2008; Kong et al., 2014) sources have been structurally characterized and were found to bear strong resemblance to another family of the Asp nucleophile containing α/β hydrolase enzymes, the haloalkane dehalogenases (Franken et al., 1991; Novak et al., 2014). Many α/β EHs are monomers, however dimers (Reetz et al., 2009; Zhou et al., 2014; cyanobacterium *Nostoc* sp. EH, PDB 3QYJ) and tetramers (Nardini et al., 1999) were also observed. Structural features extending the classical α/β fold are present in fungal and insect EHs (Reetz et al., 2009; Zhou et al., 2014) and soluble dimeric mammalian EHs contain additional domains (Argiriadi et al., 1999; Pilger et al., 2015).

This paper presents the results obtained in the search of EHs of the α/β hydrolase fold class within the metagenomes of samples collected in hot terrestrial environments, which was part of the EU FP7 collaborative project HotZyme (www.hotzyme.com). A bio-informatics search has been carried out from metagenomic samples from a variety of hot springs. Two open reading frames (ORFs) showing good similarity with known EHs have been found in samples collected in Russia and China at 46° and 65°C respectively. The two proteins have been successfully over-expressed in *Escherichia coli* and have been characterized both biochemically and structurally. It has been possible to rationalize the differences observed in their substrate specificity using information obtained from the high resolution X-ray structures.

## Materials and methods

### Chemicals

Diols, racemic and enantiopure epoxides and rhamnose were purchased from Sigma-Aldrich (USA) or Alfa Aesar (Germany). Tryptone and yeast extract were from Sigma-Aldrich (USA). All other reagents were of analytical grade and commercially available.

### Analytical methods

Gas-chromatographic (GC) analyses to determine enantiomeric excesses of epoxides, diols and conversions were performed on a AGILENT 6850 (Network GC System) gas chromatograph equipped with a chiral capillary column (MEGA DEX DAC-BETA, Italy), having 0.25 mm-diameter, 25 m length and 0.25 μm-thickness, and with a Flame Ionization Detector (FID). The stereochemical outcome of the transformations was expressed as enantiomeric excess (*e.e*.) of the major enantiomer or as enantiomeric ratio (*E*) (Chen et al., 1982).

Analytical conditions, derivatization procedures and retention times of compounds (**1**, **2**), and (**4**)-(**6**) were previously reported (Ferrandi et al., 2015b). For details, see also Figure S5 (Supplementary Materials).

For GC analysis of compound (**3**), the column temperature was raised from 40° to 100°C at 5°C/min, then from 100° to 110°C at 1°C/min and finally from 110° to 200°C at 10°C/min at a 2 ml/min flow rate. Under these conditions retention times were: (**3**), 5.26′; (2*S*,3*S*)-diol, 16.0′; (2*R*,3*R*)-diol, 16.5′. The stereochemical configuration was determined using the commercially available standard (2*R*,3*R*)-diol.

The CD spectra were recorded on a Jasco J-1100 spectropolarimeter interfaced to a personal computer for data collection and manipulation and equipped with a thermostatically controlled cell holder. The spectropolarimeter was calibrated with a D-10-camphorsulfonic acid solution.

The far-UV CD analysis was carried out with purified protein samples dissolved in degassed water (0.15 mg/ml final concentration) in quartz cuvettes with 0.1 cm path length. Spectra were recorded in the range between 180 and 250 nm at 20° or 95°C.

For the determination of apparent melting temperature (T_M_), unfolding transitions as a function of temperature were monitored by the CD signal at 193 nm or 220 nm varying the temperature as follows:

#### Sibe-EH sample

20°C up to 40°C at 5°C/min_ data pitch each 2°C, hold 30″

40°C up to 70°C at 2.5°C/min_data pitch each 0.5°C, hold 30″

70°C up to 95°C at 5°C/min pitch data each 2°C, hold 30″

#### CH65-EH sample

20°C up to 70°C at 5°C/min_ data pitch each 2°C, hold 30″

70°C up to 90°C at 2.5°C/min_data pitch each 0.5°C, hold 30″

90°C up to 95°C at 5°C/min pitch data each 2°C, hold 30″

### *In silico* screening of metagenomes assemblies

The HotZyme assemblies available on the Galaxy based platform ANASTASIA (Automated Nucleotide Aminoacid Sequences Translational platform for Systemic Interpretation and Analysis) (Menzel et al., 2015) were analyzed by using the ORF finder “getorf” program (http://emboss.bioinformatics.nl/cgi-bin/emboss/getorf) and the resulting ORFs were aligned with the query sequences [GenBank CAA73331.1 (*Agrobacterium radiobacter*-EH), CAB59813.1 (*Aspergillus niger*-EH), ACO95125.1 (kau2-EH), 2CJP_A (*Solanum tuberosum*-EH)] using the program LAST (http://last.cbrc.jp/).

### Enzyme cloning into the pJet vector

The genes coding for Sibe-EH and CH65-EH were amplified using the primers F1/R1 and F2/R2, respectively (see Table S1), and the DNA extracted from the corresponding environmental samples as a. template (Ferrandi et al., 2015b; Menzel et al., 2015). The PCR amplification was carried out on 50 μL reaction mixtures containing 100 ng of metagenomic DNA, primers (1 μM each), dNTPs (0.2 mM each), 4 U of XtraTaq Pol and 5 μL of the XtraTaq buffer (both from Genespin, Italy). The PCR conditions were as follows: 95°C for 3 min, followed by 40 cycles at 94°C for 30 s, 50–55°C (according to the primer annealing temperatures) for 30 s, 72°C for 1 min, and then 72°C for 10 min. The genes were cloned in the pJet vector using the CloneJET PCR Cloning Kit (Thermo Scientific, United States) and the resulting plasmids pJetSibe-EH and pJetCH65-EH were transformed into *E. coli* TOP 10 (Invitrogen, United States) using standard techniques (Sambrook and Russell, 2001). The two plasmids were purified by using the HiSpeed Plasmid Midi Kit from Qiagen (Germany) and the cloned PCR amplicons were sequenced on both strands by Bio-Fab Research (Italy) using the primers F8/R8 (Table S1).

### Enzyme expression and purification

Sibe-*eh* and CH65-*eh* genes were amplified using pJetSibe-EH and pJetCH65-EH as template, respectively. Primers F3/R3 and F4/R4 (Table S1) suitable for the cloning of EH genes in the pRham expression vector (*Expresso* Rhamnose Cloning and Protein Expression kit, Lucigen), were used for PCR gene amplifications under the PCR conditions described above. The PCR products were cloned in the pRham vector giving pRhamSibe-EH and pRhamCH65-EH plasmids respectively, and transformed in *E. coli* 10G. pRhamSibe-EH and pRhamCH65-EH were purified using the HiSpeed Plasmid Midi Kit from Qiagen (Germany) and subsequently transformed in *E. coli* BL21 codon plus RIPL competent cells (Agilent Technologies, United States) according to manufacturer instructions.

The transformants obtained were grown in LB supplemented with 30 μg mL^−1^ kanamycin (LB_kan30_) medium (50 mL) overnight and then inoculated in 0.5 L LB _kan30_ at 37°C and 220 rpm. When the OD_600_ reached 0.3–0.6, gene expression was induced by the addition of 5 mL 20% rhamnose solution (w/v in water) and the culture was maintained at 30°C for 24 h. Then cells were harvested by centrifugation (5,000 rpm for 30 min), resuspended in 20 mL of wash buffer (20 mM potassium phosphate (KP) buffer, pH 7.0, 500 mM NaCl, 20 mM imidazole) and disrupted by sonication. In the case of CH65-EH, another expression trial was carried out lowering the culture incubation temperature to 17°C after induction of protein expression and the cells were harvested after 72 h.

Recombinant EHs were subsequently purified using a Nickel Sepharose 6 Fast Flow agarose resin (Ni-NTA) (GE-Healthcare, Italy) as follows. The cell extract, recovered by centrifugation (10,000 rpm for 30 min) after cell lysis, was incubated with the Ni-NTA resin for 1 h at 4°C with mild shaking and loaded onto a glass column (10 × 110 mm). The resin was then washed with 10 mL of wash buffer and His-tagged EHs were eluted using a 3 step gradient (10 mL washing buffer containing 100, 200, and 300 mM imidazole, respectively) and dialyzed against 20 mM KP buffer, pH 7.2, at 4°C. The protein content was measured using the Bio-Rad Protein Assay according to the Bradford method and the protein purity was verified by SDS-PAGE analysis (10% T, 2.6% C).

Due to unsatisfactory results obtained using the metagenome-derived CH65-*eh* gene, the codon-optimized CH65-*eh* gene was synthesized and cloned into the pUC57 vector obtaining pUC-CH65-EHopt by BaseClear (Leiden, The Netherlands). The CH65-*eh* codon optimized gene was then amplified using the primers F5/R5 (Table S1) and pUC-CH65-EHopt as a template for the suitable cloning into the pETite vector. Gene amplification was carried out under the PCR conditions described above and PCR products were cloned into the pETite vector and transformed into the *E. coli* Hi control 10 G using the *Expresso* T7 Cloning and Expression kit from Lucigen. The resulting plasmid pETiteCH65-EHopt was purified and transformed into *E. coli* BL21(DE3) containing the plasmid pG-Tf2 (Takara Bio Inc., Kyoto, Japan) that allows co-expression of the target protein with the chaperone proteins GroES and GroEL.

Transformants were grown overnight in 50 mL of LB medium supplemented with 30 μg mL^−1^ kanamycin and 20 μg mL^−1^ chloramphenicol (LB_kan30cam20_) and then inoculated in 0.5 L LB_kan30cam20_ medium containing 5 ng μL^−1^ tetracycline for induction of chaperon proteins at 37°C. Subsequently, when the cell density reached OD_600_ 0.4–0.6, the gene expression was induced by the addition of 1 mL solution of IPTG (1 M in water) and the culture was incubated at 30°C. The cells were harvested by centrifugation (5,000 rpm for 30 min) 24 h after induction, resuspended in 20 mL of wash buffer (see above), and lysed by sonication. The CH65-EH was purified as described above.

### Enzyme characterization

Hydrolysis of epoxides (**1**)-(**6**) was performed with the purified Sibe-EH [0.25 mg for the hydrolysis of (**1**), (**2**), (**4**), 0.5 mg for the hydrolysis of (**3**), 0.05 mg for the hydrolysis of (**5**), 0.125 mg for the hydrolysis of (**6**)] or with the purified CH65-EH [0.5 mg for the hydrolysis of (**1-4**) and (**6**), 0.01 mg for the hydrolysis of (**5**)] in 1 mL 25 mM KP buffer, pH 8.0, 10% (v/v) CH_3_CN, containing 10 mM substrate, at 20°C (SibeEH) or 45°C (CH65-EH). At scheduled times, samples (50 μL) were extracted with an equal volume of a 0.025 mg/mL benzophenone solution in AcOEt in the presence of saturating NaCl and analyzed by chiral GC analyses. The substrates and products peak area were normalized to the internal standard benzophenone and concentrations were calculated using calibration curves obtained with authentic substrate/product standards (2.5–20 mM). One unit of activity (U) is defined as the enzyme activity that hydrolyzes 1 μmol of substrate per min under the assay conditions described above.

The evaluation of the influence of temperature on Sibe-EH activity and CH65-EH were determined by assaying the hydrolysis of (**2**) at a temperature ranging from 20° to 90°C.

The thermal stability was evaluated by incubating purified Sibe-EH or CH65-EH samples at different temperatures (20–70°C) for 3 h, then assaying the residual activity using (**2**) as a substrate under the above described conditions.

### Crystallization and structure solution

Prior to crystallization both the purified Sibe-EH and CH65-EH were applied to a calibrated Superdex 200 HiLoad 16/60 size exclusion column (GE Healthcare) and were eluted with one column volume of a buffer of 25 mM Tris-HCl, pH 7.5, 0.1 M NaCl at 1.0 ml/min.

The Sibe-EH and CH65-EH were then concentrated to ~15 mg/ml and ~30 mg/ml respectively using a 10 kDa membrane Vivaspin (Vivaproducts) and microbatch crystallization trials were set up using an Oryx6 crystallization robot (Douglas Instruments) using the JCSG Screen+™, SG1™ Morpheus™ (Molecular Dimensions) protein crystallization screens. The droplet contained a 50:50 ratio of protein solution to screen and was covered with Al's oil (50:50 mix of silicon and paraffin oils) before being stored at 20°C.

Sibe-EH native crystals appeared within 1 week in the majority of the conditions of the Morpheus screen. The crystals were harvested straight from the crystallization droplet and plunged into liquid nitrogen. Preliminary data were collected to 2.4 Å resolution at 100 K on the Diamond beamline I03. Further crystals grown from 0.1 M Sodium HEPES, MOPS buffer pH 7.5, 12.5% v/v methyl pentanediol (MPD), 12.5% w/v PEG 1,000, 12.5 % w/v PEG 3350 diffracted to a higher resolution of 1.7 Å in the same space group on the Diamond beamline I04 and these data were used for final refinement.

The CH65-EH native crystals appeared within 1 week. The best crystals grew in 0.2 M magnesium formate dehydrate, 100 mM sodium HEPES pH 7.5 20% w/v PEG 3350 and were cryocooled in liquid nitrogen using a cryoprotectant consisting of 0.2 M magnesium formate dehydrate, 0.1 M sodium chloride, 0.1 M sodium HEPES pH 7.5, 16% w/v PEG 3350, 30% v/v PEG 400.

The Sibe-EH and CH65-EH crystals diffracted to 1.6 Å and 1.4 Å, respectively on the beamlines I03 and I04 at the Diamond Synchrotron light source (Didcot, United Kingdom) at 100 K in a stream of gaseous nitrogen using a Pilatus detector (Dectris). Data were processed and scaled using XDS (Kabsch, 2010) and AIMLESS (Evans and Murshudov, 2013) in the Xia2 pipeline (Winter et al., 2013). All further data and model manipulations were carried out using the CCP4 suite of programs (Winn et al., 2011). The Sibe-EH crystals belonged to the spacegroup C222_1_ with unit cell parameters *a* = 41.2, *b* = 84.2, *c* = 157.5Å, α = β = γ = 90° and the CH65-EH crystal belonged to the spacegroup C2 with unit cell parameters *a* = 163.9, *b* = 46.2, *c* = 73.9 Å, α = γ = 90° β = 106.9°.

The Sibe-EH structure was solved using the epoxide hydrolase from *Bacillus megaterium* (31% sequence identity; PDB: 4INZ; Kong et al., 2014) using Molrep (Vagin and Teplyakov, 2010) with its sequence-based model modification option (Lebedev et al., 2008). The rotation function gave a clear peak of 8.7 σ with a background of 4.9 σ or lower and a translation peak of 8.2 σ with a background of 3.5 σ. The resulting structure was subjected to refinement in REFMAC5 (Murshudov et al., 2011) and rebuilding in COOT (Emsley et al., 2010; **Table 2**). The structure of CH65-EH was solved by the molecular replacement pipeline MORDA (Lebedev and Vagin, 2015) with PDB 4INZ (35% sequence identity) being the best model.

### Molecular modeling

For both Sibe-EH and CH65-EH structures, the chain A of each structure was used in the ligand docking experiments. The protein structures were initially prepared using the Protein Preparation Wizard (Sastry et al., 2013) in the Schrödinger Maestro release 2017-1 (Schrödinger). All rigid (rigid protein, flexible ligand) and flexible (flexible protein side chains, flexible ligand) dockings were carried out using AutoDock Vina version 1.1.2 (Trott and Olson, 2010). The receptors and ligands PDBQT files, required by the docking program, were generated using AutoDockTools (Morris et al., 2009). In rigid docking experiments, the two cavities around the Asp101, Tyr148, and Tyr209 for CH56-EH and Asp102, Tyr150, and Tyr209 for Sibe-EH were chosen as the binding sites of the ligands. In all rigid docking experiments, exhaustiveness of the global search was set to 100, maximum number of binding modes was set to 20, and the energy range was set to 10.

In flexible docking experiments, side chains of residues surrounding the potential substrate binding site of the two enzymes were set to be flexible. These include the residues Phe34, Asp101, Trp102, Tyr148, Tyr209, Ala243, Ile244, His270 for CH65-EH, and Asp102, Trp103, Tyr150, Tyr209, Asp249, Ala251, Leu252, His277 for Sibe-EH. The ligand binding sites for the AutoDock Vina docking experiments were chosen to be large enough to include all of the flexible residues and the two binding site cavities. In all rigid docking experiments, exhaustiveness of the global search was set to 8, maximum number of binding modes was set to 20, and the energy range was set to 10.

Docking results were visualized and analyzed using the ViewDock tool in UCSF Chimera (Pettersen et al., 2004) and in COOT (Emsley et al., 2010). The best ligand docking poses were chosen according to their binding affinities as reported by AutoDock Vina, and also according to their configurations in relation to the binding site residues.

The programs CAVER (Chovancova et al., 2012; Kozlikova et al., 2014; Pavelka et al., 2015) and UCSF Chimera were used for the analysis and visualization of the active sites tunnels and surfaces. For the calculation and visualization of tunnels and surfaces using CAVER and UCSF Chimera programs, probes with a radii of 1.1 and 1.4 Å were used. Additional ligand and transient state models were generated by JLIGAND (Lebedev et al., 2012).

## Results and discussion

### Discovery of new EH homologs

When the metagenomic ORFs identified in the HotZyme project were aligned with the amino acid sequences of the well characterized α/β EHs from *Agr. radiobacter, B. megaterium, Asp. niger*, and *S. tuberosum*, as well as the metagenome derived enzyme Kau2-EH, two ORFs showed good similarity. These were found in the metagenomes of samples collected at 46° and 65°C in the West Siberian Plain of Russia (Tomsk sample) and in the Yunnan region of China (Ch2-EY65S sample), respectively, at around neutral pH. It is worth noting that the new EH α/β class homologs were identified in the same two environments, where we had previously isolated the LEHs which belong to a different structural family (Ferrandi et al., 2015b). These environments were characterized by moderate temperature and neutral pH. As in the case of the LEH search, no EH homologs were found in samples collected at higher temperatures, suggesting that under extreme conditions EHs are not required in hyper-thermophilic organisms since the epoxides used as substrates for these enzymes would spontaneously hydrolyse. A detailed description of the Tomsk and Ch2-EY65S sampling sites has been previously reported (Ferrandi et al., 2015b; Menzel et al., 2015).

The ORF found in the Chinese metagenomic DNA sample (CH65-*eh*) consists of 879 nucleotides and encodes for a protein (CH65-EH) of 293 amino acids. According to the BLAST analysis, this protein shows the highest similarity to a α/β hydrolase from *Nitrosomonas marina* (GenBank WP_090634503.1) (50% identity and 99% query cover sequence at the deduced amino acid level) and presents 35% identity with the EH from *B. megaterium* (Zhao et al., 2011).

**Figure 1:**
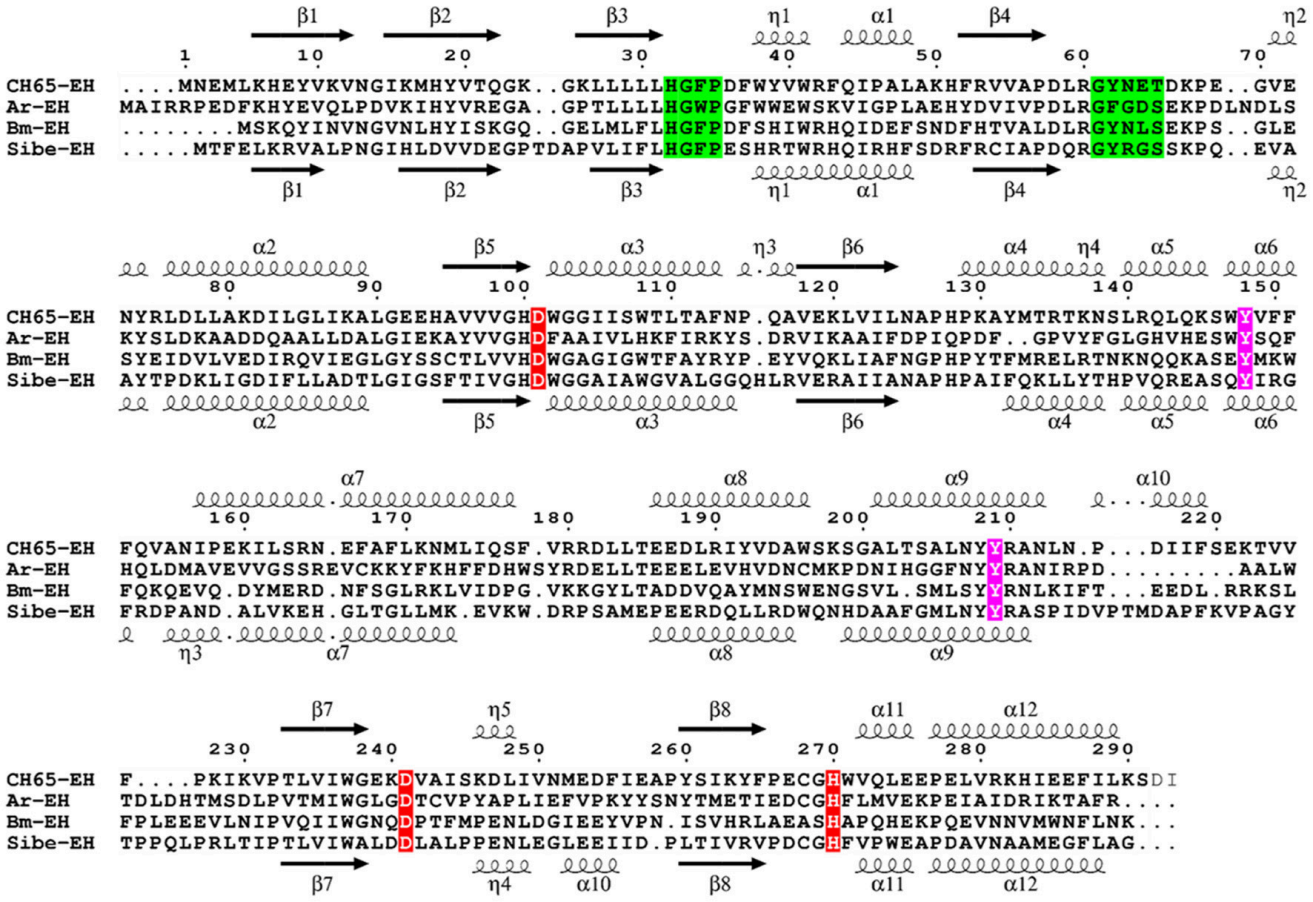
Multiple sequence alignment of Sibe-EH and CH65-EH with *Agr. radiobacter* EH (*Ar*-EH) and *B. megaterium* EH (*Bm*-EH). Arrows indicate β-strands, and helical curves denote α-helices of the structure of CH65-EH above and Sibe-EH below. The three putative active site residues (D/H/D) are marked in red. Ring opening tyrosines are shown in purple. The conserved HGXP and GXSmXS/T motifs are highlighted in green. The figure was prepared with ESPript3 (Robert and Gouet, 2014).

The ORF found in the Russian metagenome (Sibe-*eh*) is 891 nt long and encodes for a protein (Sibe-EH) of 297 amino acids that has 30% identity with the CH65-EH protein sequence. The closest relative of Sibe-EH is an α/β hydrolase from *Porphyrobacter* sp. LM 6 (GenBank WP_083234595.1) showing 92% identity and 100% query cover sequence at the deduced amino acid level. Such a high identity suggests that the organism that expresses Sibe-EH may be a bacterium that belongs to the genus *Porphyrobacter*. When compared to other functionally characterized bacterial EHs, the highest similarity shown by Sibe-EH was again with *B. megaterium*-EH (32% identity) (Zhao et al., 2011). A Pfam-A database analysis confirmed that the two new EH homologs belong to the α/β hydrolase superfamily. A sequence alignment with related EH homologs shows the expected conservation of the amino acids of the catalytic triad (Asp-His-Asp), as well as of the two tyrosine residues involved in the epoxide ring opening, and of the EH conserved motif HGXP (the oxyanion hole) (Figure 1; Van Loo et al., 2006). In contrast the previously reported conserved motif GXSmXS/T (where X is usually an aromatic amino acid and Sm is a small amino acid such as Gly, Ala or Cys) was only partially conserved since the basic amino acid, arginine, R64, was present in Sibe-EH and an asparagine residue, N63, in CH65-EH at the Sm position. It is worth noting that an asparagine residue is also present at this position in *B. megaterium*-EH (Zhao et al., 2011). Figure S1 shows a phylogenetic analysis for Sibe-EH and CH65-EH in relation to selected known EHs.

### Expression trials and purification of EHs

The genes CH65-*eh* and Sibe-*eh* were successfully cloned in the pRham expression vector in frame with the C-terminal His6x-tag sequence under the control of the L-rhamnose inducible promoter. A rare Codon Calculator (RaCC, http://people.mbi.ucla.edu/sumchan/caltor.html) analysis of CH65-*eh* and Sibe-*eh* (see Doc S1) revealed that both genes contained codons that are rarely recognized by tRNAs in *E. coli* which is a potential problem for good expression yields of heterologous proteins in this host. For this reason, *E. coli* BL21 codon plus RIPL, a strain containing two plasmids that encode for rare tRNAs that recognize the rare codons for Arg, Ile, Pro, and Leu, was chosen as host for the expression of CH65-*eh* and Sibe-*eh*.

The His-tagged Sibe-EH fusion protein was successfully over-expressed in soluble form in *E. coli* BL21 codon plus RIPL and purified from the cell extracts by Ni^2+^-NTA affinity chromatography with an excellent recovery yield (288 mg L^−1^) (see Figure S2).

On the contrary, very low levels of expression were observed for the His-tagged CH65-EH fusion protein using the same host. Moreover, this recombinant protein formed insoluble aggregates within the *E. coli* cells and almost no soluble protein was observed in the cell free extracts as shown by SDS-PAGE analysis. Lowering of the culture incubation temperature to 17°C after induction of protein expression did not prevent the formation of the inclusion bodies and little soluble protein was detected (data not shown).

To improve the recombinant production of CH65-EH in *E. coli*, a codon optimized CH65-*eh* synthetic gene (BaseClear) was cloned into the pETite (Lucigen) vector under the control of the strong IPTG inducible T7 promoter. Moreover, to tackle the previously observed solubility issues, the gene was expressed in *E. coli* BL21(DE3) containing the plasmid pG-Tf2 (Takara Bio Inc.) which allows the co-expression of the target protein with the chaperon proteins GroES and GroEL. This improved the expression and the solubility of the His-tagged CH65-EH (see Figure S3) resulting in a yield of 50 mg L^−1^ of pure enzyme after purification by Ni^2+^-NTA chromatography.

### Characterization of EHs

**Figure 2:**
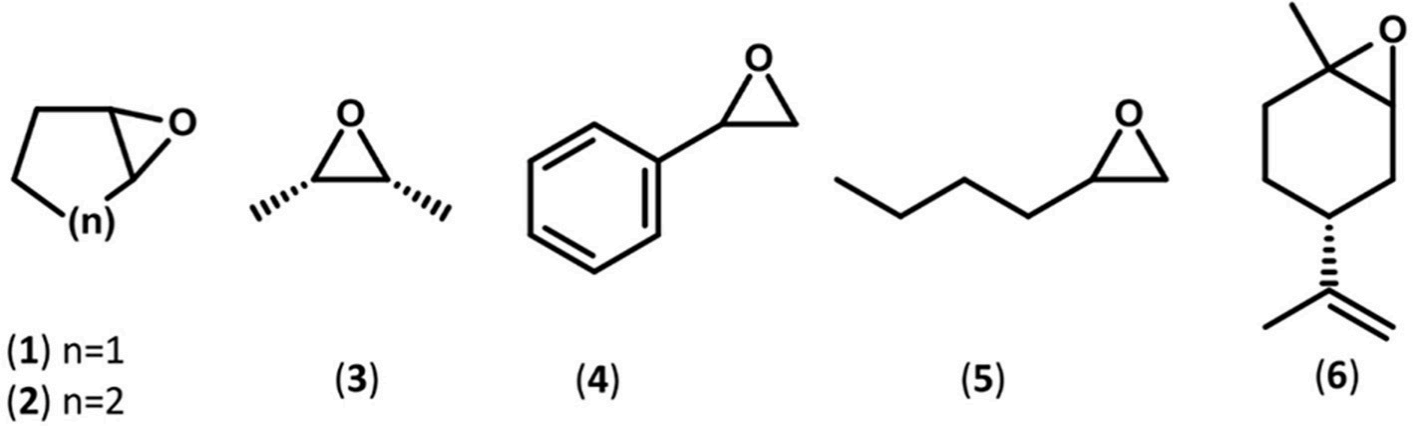
Schematic representations of the substrates used in this study.

**Figure 3:**
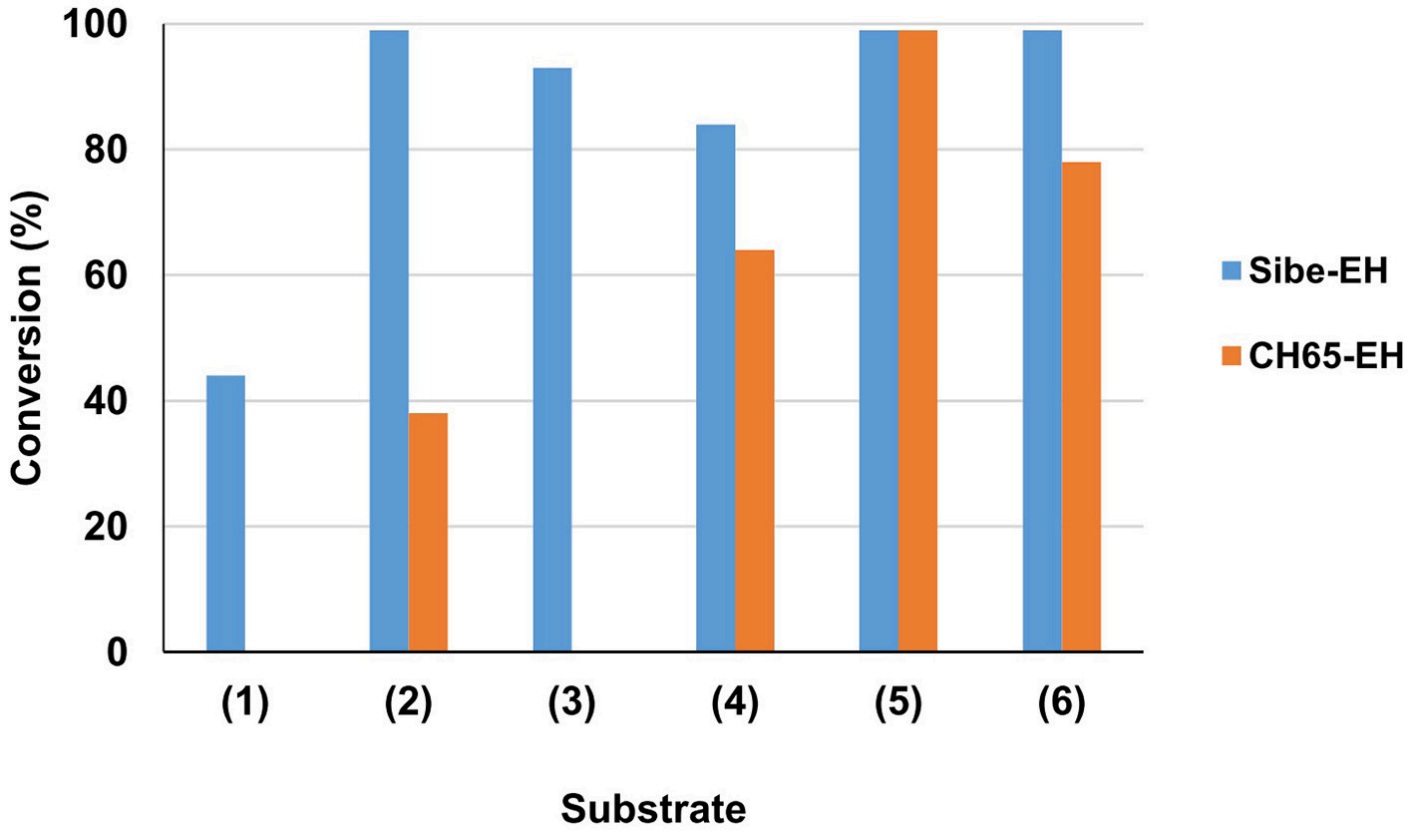
Preliminary investigation of EHs substrate specificity. Reactions were carried out in 25 mM KP buffer, pH 8.0, 10% (*v*/*v*) CH_3_CN containing 0.5 mg of purified EH, 10 mM substrate (**1**–**6**) in 1 ml total volume, at 20°C. Substrate conversion was evaluated by GC analysis after 24 h.

The substrate specificity of the novel EHs was carried out by evaluating the conversion of a set of structurally different epoxide substrates (Figure 2) after 24 h under standard reaction conditions (0.5 mg mL^−1^ of purified EHs, 20°C). The Sibe-EH was active on all tested substrates resulting in conversions > 99% for substrates (**2**), (**5**), and (**6**), around 90% for substrates (**3**) and (**4**), and around 40% for substrate (**1**) (Figure 3). The CH65-EH showed a preference for substrates (**4**) and (**6**) (conversions >60%) and in particular for substrate (**5**) (conversion > 99%), while negligible activity was shown on substrates (**1**) and (**3**).

The overall thermostability of the two novel EH enzymes was investigated by far UV Circular Dichroism (CD) spectra at temperatures of 20° and 95°C to monitor any conformational changes of the enzyme secondary structures. This showed that at 95°C the far UV CD spectrum of Sibe-EH (see Figure S4) is significantly different from the spectrum recorded at 20°C. In contrast, the comparison of the far UV CD spectrum of CH65-EH at 95°C and at 20°C suggests only partial unfolding at 95°C. The higher thermostability of CH65-EH in relation to Sibe-EH was subsequently confirmed by determining the apparent T_M_ of the two enzymes by the thermal shift CD analysis. The Sibe-EH showed the behavior of a moderately thermophilic enzyme with an apparent T_M_ of 55°C, while CH65-EH showed higher thermal stability with an apparent T_M_ of 85°C, which is, to our knowledge, the highest T_M_ recorded for a wild-type EH to date.

**Figure 4:**
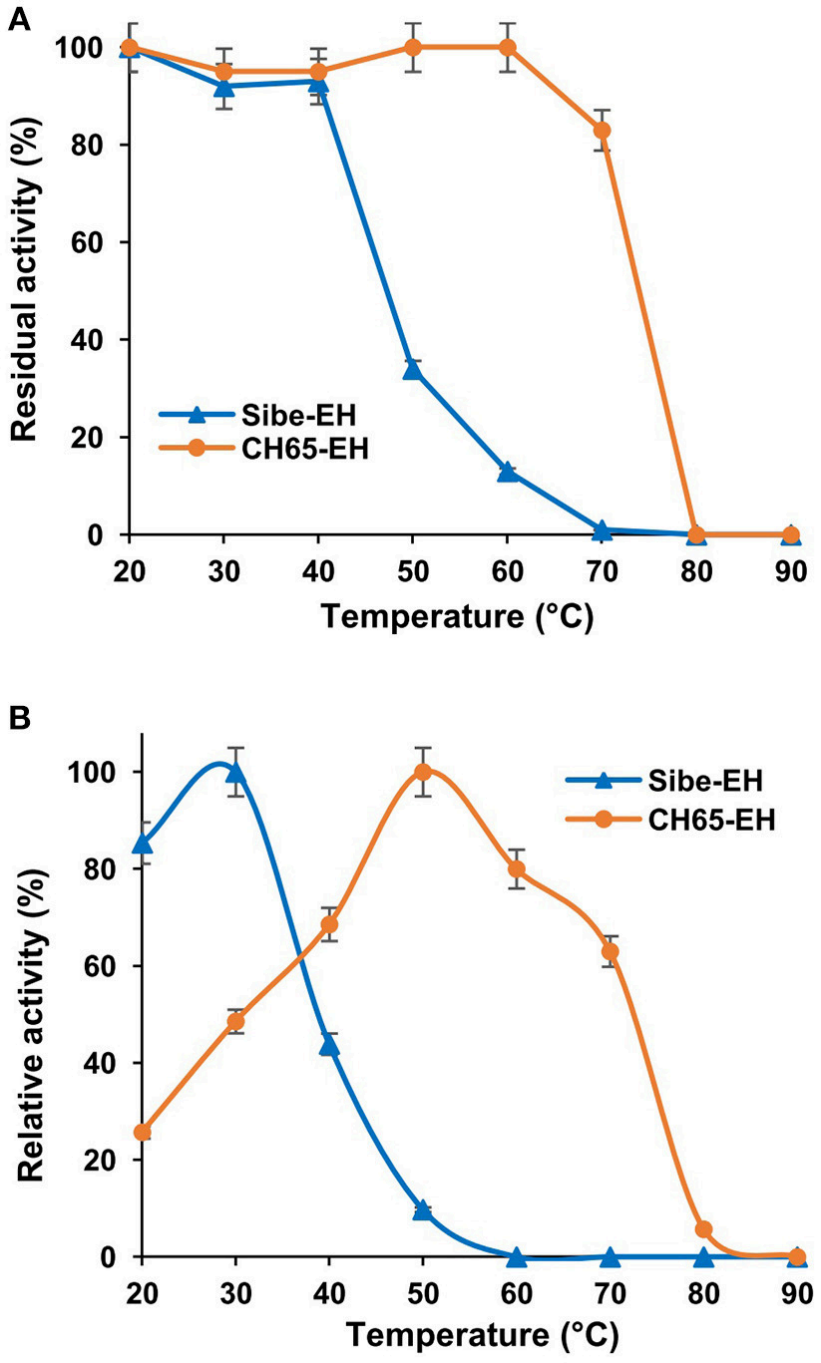
**(A)** The effects of temperature on stability of the Sibe-EH (blue triangles) and CH65-EH (orange circles) **(B)** The effects of temperature on enzymatic activity of the Sibe-EH (blue triangles) and CH65-EH (orange circles).

When the thermostability of Sibe-EH and CH65-EH was further investigated by incubating the enzymes for 3 h at temperatures ranging from 20° to 90°C and then assaying the hydrolysis of compound (**2**) by the two heat-treated enzymes (Figure 4A) the results were in agreement with those obtained from CD and thermal shift analysis. The CH65-EH was significantly more thermostable than Sibe-EH, with no decrease of activity after incubation at temperatures of 60°C and retaining about 80% of the initial activity after thermal treatment at 70°C, while Sibe-EH was relatively stable at temperatures below 40°C and was able to retain 10% of initial activity after incubation at 60°C.

The temperature dependence of EH activity of both enzymes was evaluated by carrying out the hydrolysis of compound (**2**) at temperatures ranging from 20 to 90°C, pH 8.0. As shown in Figure 4B, Sibe-EH displayed an optimum temperature at 30°C and retained more than 40% of activity at 40°C, while CH65-EH showed the highest activity at temperatures around 50°C and retained more than 60% of activity at 70°C.

Subsequently, considering the different optimal temperatures, the specific activity and selectivity of the recombinant EHs was assessed at 30° and 45°C for Sibe-EH and CH65-EH, respectively, using chiral GC analyses of the hydrolysis reactions of the epoxide substrates (**1**)-(**6**) (Figure 2, Table 1).

**Table 1:** Substrate scope and selectivity of Sibe-EH and CH65-EH.

**Substrate**	**Sibe-EH ^a^**		**CH65-EH ^a^**	
	**Specific activity (U·g^−1^)**	**Selectivity^b^**	**Specific activity (U·g^−1^)**	**Selectivity^b^**
(**1**)	12	*ee*_p_ (%) = 66 (*1R,2R*)	n.d.^c^	–
(**2**)	80	*ee*_p_ (%) = 80 (*1R,2R*)	68	*ee*_p_ (%) = 85 (*1R,2R*)
(**3**)	107	*ee*_p_ (%) ≥ 99% (*2R,3R*)	n.d.^c^	–
(**4**)	71	*E* = 1	87	*E* = 1
(**5**)	1730	*E* = 5 (*R*)	6,359	*E* = 2 (*R*)
(**6**)^d^	1628 n.d.^c^	(*trans*) (*cis*)	95 34	(*trans*) (*cis*)

a *Reactions catalyzed by Sibe-EH were performed at 30°C, while reactions catalyzed by CH65-EH were performed at 45°C*.

b *selectivity is indicated as enantiomeric excesses of the products (ee_p_) in the case of meso-epoxides (**1**–**3**), and E values in the case of the racemic substrates (**4**–**5**)*.

c *n.d., not detected*.

d *substrate (**6**) is a commercially available mixture of cis and trans isomers of (4R)-limonene-1,2-epoxide*.

When investigating the stereoselective hydrolysis of the *meso*-epoxides (**1**)-(**3**), both Sibe-EH and CH65-EH hydrolysed compound (**2**) with comparable velocity and stereoselectivity (Table 1). However, compounds (**1**) and (**3**) were hydrolysed only by Sibe-EH, which additionally showed an excellent stereo-selectivity [>99% enantiomeric excess (*ee*_p_)] for the synthesis of the (2*R*,3*R*)-diol from the achiral starting compound (**3**). Although highly enantioselective variants have been recently obtained by protein engineering (van Loo et al., 2009; Zheng and Reetz, 2010), to our knowledge this is the best result that has been obtained with a wild-type EH for the enantioselective hydrolysis of compound (**3**) to give the corresponding (2*R*,3*R*)-diol. The best results that had been obtained previously were with whole cells of *Rhodotorula glutinis* CIMW 147 which achieved the formation of the (2*R*,3*R*)-diol with 90% *ee*_p_ (Weijers, 1997).

The specific activity and enantioselectivity of the two enzymes were also compared in the kinetic resolution of compounds (**4**)-(**5**). As shown in Table 1, compound (**4**) was hydrolysed by both enzymes with similar velocity and no selectivity, while CH65-EH hydrolysed compound (**5**) four times faster than Sibe-EH, with both enzymes showing very low enantio-preference for the (*R*)- enantiomer. The preference showed by both enzymes for the hydrolysis of compound (**5**) compared to compound (**4**) [the specific activity for compound (**5**) is two orders of magnitude higher than the specific activity for compound (**4**)] could be due to the absence of the aromatic ring in substrate (**5**).

Finally, the two new EHs were also tested for the hydrolysis of the mixture of *cis* (1*R*,2*S*,4*R*) and *trans* (1*S*,2*R*,4*R*) isomers of (+)-limonene oxide [compound (**6**), i.e., the natural substrate of limonene EHs (LEHs)]. Surprisingly, although EHs belonging to the α/β superfamily and LEHs represent two completely distinct enzyme classes with different structures and catalytic mechanisms, both the new EHs were able to efficiently hydrolyse compound (**6**) forming (1*S*,2*S*,4*R*)-limonene-1,2-diol as the only product through the same enantio-convergent process previously described for LEHs (Van Loo et al., 2006). Moreover, Sibe-EH showed an almost exclusive preference for the *trans* isomer, which was hydrolysed in the first hour of the reaction leaving the *cis* isomer untouched and was even more efficient than the previously described Tomsk-LEH and CH65-LEH in the enantioselective hydrolysis of (**6**). In fact, the specific activity for the hydrolysis of the *trans* isomer (1,628 U·g^−1^, Table 1) was significantly higher than those shown by LEHs (Tomsk-LEH: 220 U·g^−1^; CH55-LEH: 400 U·g^−1^) (Ferrandi et al., 2015b).

This is the first time that the hydrolysis of compound (**6**) has been described to be catalyzed by epoxide hydrolases belonging to the α/β superfamily. In fact, to our best knowledge, hydrolysis of this compound has only been verified using either a LEH or whole cell biotransformations such as cultures of *R. glutinis* CIMW147 (Weijers, 1997) without investigating whether the EH responsible for the hydrolysis of (**6**) was a LEH or not.

### X ray structure quality

**Figure 5:**
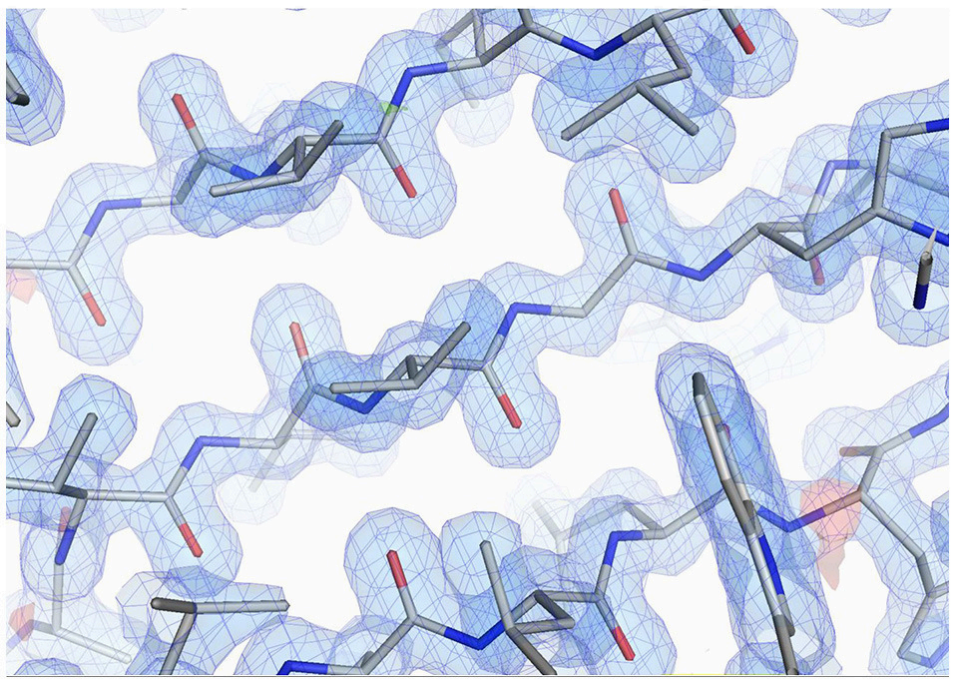
The quality of the electron density from the CH65-EH in the β-sheet region. The 2Fo-Fc map is shown in blue at a level of 1.3 σ. The Fo-Fc map is shown at 3.0 σ level (green) and −3.0 σ level (red). Figure was prepared using PyMOL (DeLano, 2002).

The structures of both Sibe-EH and CH65-EH have been determined and refined to a high resolution of 1.6 and 1.4 Å, respectively (Figure 5 shows the quality of the CH65-EH electron density and crystallographic values are shown in Table 2). All residues of Sibe-EH plus one His residue belonging to the C-terminal His tag have been modeled into the electron density. Residues 1–2 and 231–233 of Sibe-EH have been modeled with alternative conformations of the main chain. The amino acids Pro36 and Pro266 are in the *cis*- conformation in the Sibe-EH structure. Residues Arg64, His117 and the catalytic Asp102 of Sibe-EH are in a generously allowed conformation regions of the Ramachandran plot as defined by PROCHECK (Laskowski et al., 1993). Residues 2–293 were modeled in monomer A and 2–291 in monomer B out of the total 293 in the CH65-EH sequence with the C-termini having different conformations in monomers A and B beyond residue Ile288. The amino acid residues 253–255 and 157–258 in monomer A and 12–14 in monomer B of CH65-EH were modeled with alternative conformations of the main chain. The Pro35 is in the *cis*- conformation in both monomers of CH65-EH. The residues Asn63 and Asn156 are Ramachandran plot outliers in both monomers of CH65-EH with Phe37 and the catalytic Asp101 of both monomers in the generously allowed regions of the Ramachandran plot.

**Table 2:** Summary of data processing and refinement statistics.

	**Sibe-EH**	**CH65-EH**
Diffraction source	I04, I03, Diamond	I04, I03, Diamond
Wavelength (Å)	0.9795	0.9795
Space group	C222_1_	C2
*a, b, c* (Å)	41.20, 84.18, 157.45	163.94, 46.22, 73.87
α, β, γ (°)	90.0, 90.0, 90.0	90.0, 106.9, 90.0
Resolution range (Å)	39.36–1.60 (1.64–1.60)^a^	39.53–1.39 (1.41–1.39)
Number of unique reflections	35.698 (2,070)	104,819 (4,187)
Completeness (%)	97.4 (78.3)	98.2 (79.0)
Average redundancy	6.0 (4.1)	3.2 (2.5)
<*I*/σ(*I*)>	9.5 (1.3)	15.6 (1.2)
R_sym_ (%)^b^	10.6 (101.5)	3.5 (77.0)
${\text{CC}}_{1/2}^{\text{c}}$	0.996 (0.516)	99.9 (48.2)
Overall *B* factor from Wilson plot (Å^2^)^c^	26.9	24.9
*R_*fact*_ (%)*	16.2	17.3
*R*_free_ *(%)*	19.8	20.4
Refined protein atoms	2,635	5,401
Refined solvent atoms	386	645
Average B factor (Å^2^)		
Protein	19.8	23.5
Solvent	37.1	35.5
R.m.s.d. bond lengths (Å)	0.011	0.013
R.m.s.d. bond angles (°)	1.42	1.64
Ramachandran plot analysis, residues in (%)^e^		
Most favored regions	89.8	91.2
Additional allowed regions	8.9	7.3
Generously allowed regions	1.2	0.8
Disallowed regions	0	0.8

a *Values for the highest resolution shell are given in parentheses*.

b *R_sym_ = Σ_h_Σ_i_|I_h, i_ - < I_h_>|/ Σ_h_ Σ_i_I_h, i_*.

c *CC_1/2_ is defined in Karplus and Diederichs (2012)*.

*^d^ Wilson B-factor was estimated by SFCHECK (Vaguine et al., 1999)*.

e *Ramachandran plot analysis was performed by PROCHECK (Laskowski et al., 1993)*.

### EH structure

**Figure 6:**
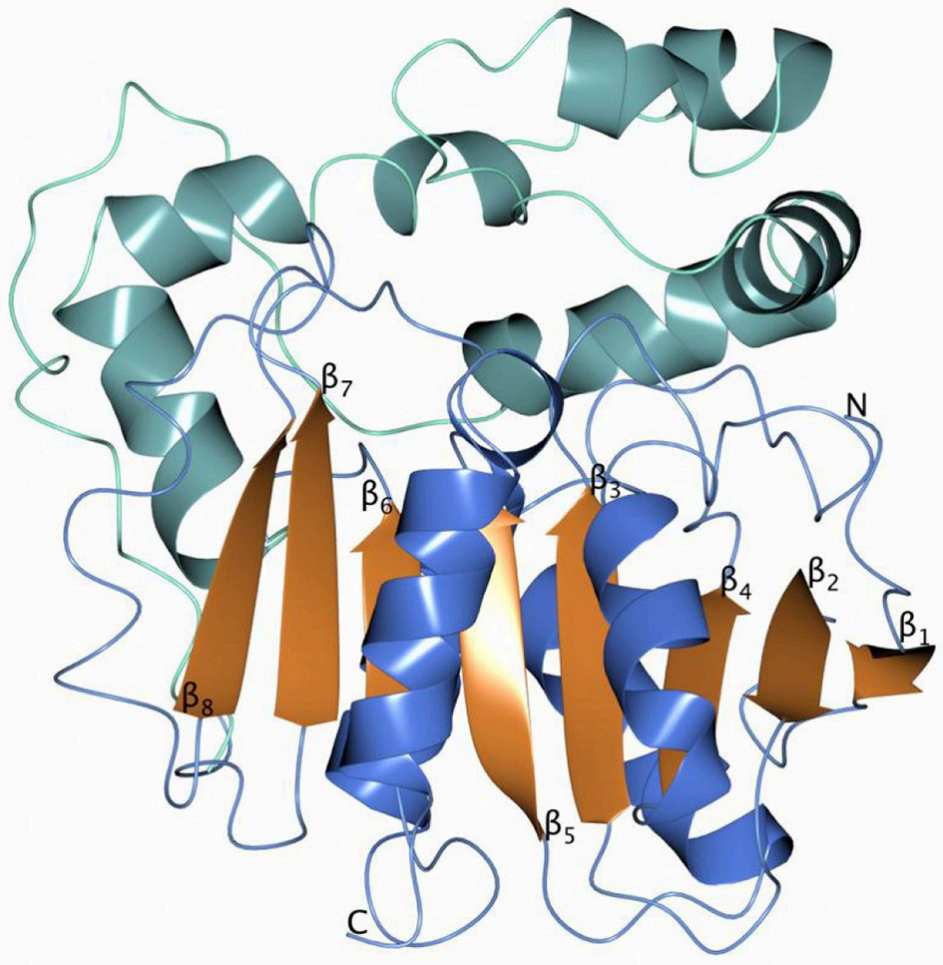
A cartoon diagram of the monomer Sibe-EH with the helices of the catalytic domain shown in blue and the helices of the cap domain in sea blue.

The overall monomer structures of both Sibe-EH and CH65-EH are similar to the structures of other α/β-hydrolases (the monomer of Sibe-EH is shown in Figure 6), consisting of a catalytic α/β domain built around an eight stranded β-sheet with connectivity 1,2,-1x,2x,1x,1x,1x and direction +-+ + + + ++ (Richardson, 1981) and an α-helical cap domain that forms a cap over the active site. The key active site residues are highly conserved with previously known epoxide hydrolase enzymes of the α/β hydrolase fold family such as the *B. megaterium* EH (Kong et al., 2014) which shares 35% amino acid sequence identity with both EHs.

**Figure 7:**
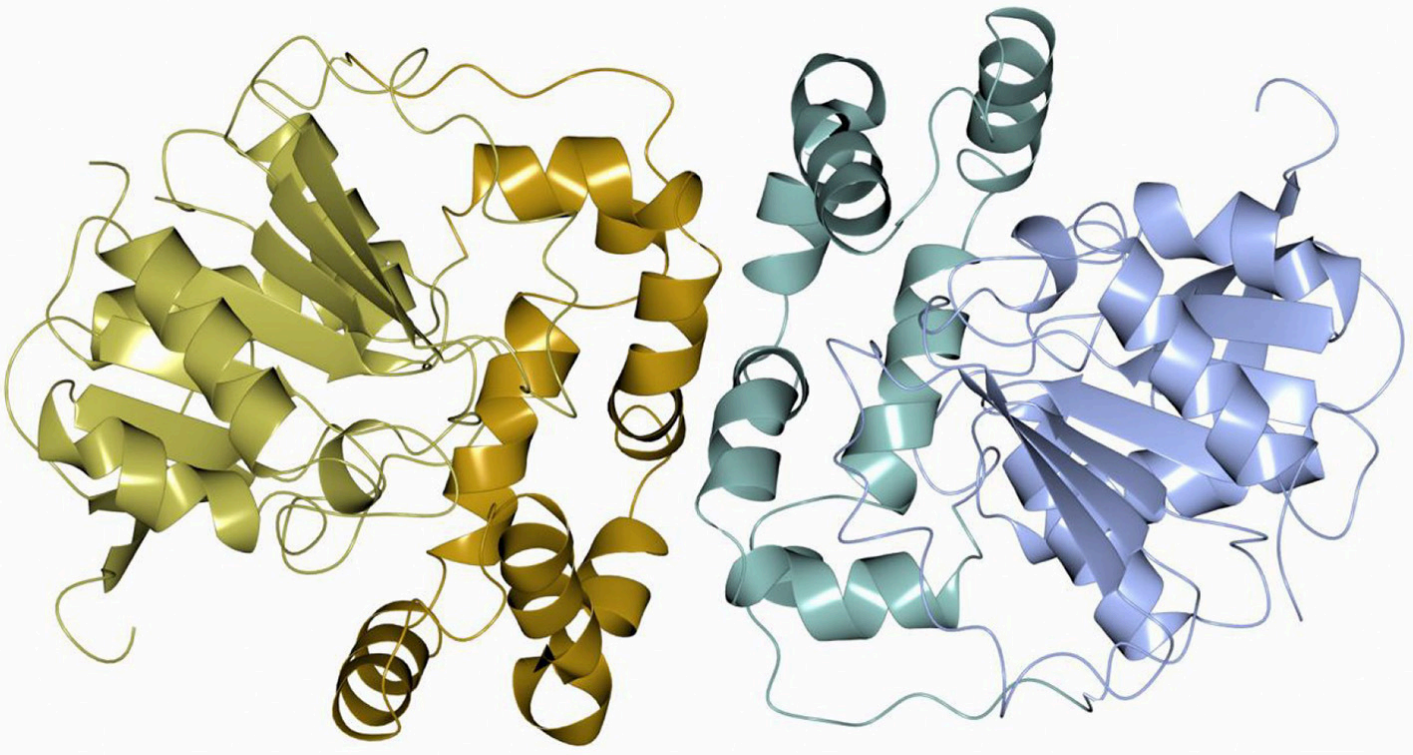
A cartoon diagram of the dimer of CH65-EH viewed along the molecular dyad. The cap domains that form the interaction within the dimer are shown in different colors.

**Figure 8:**
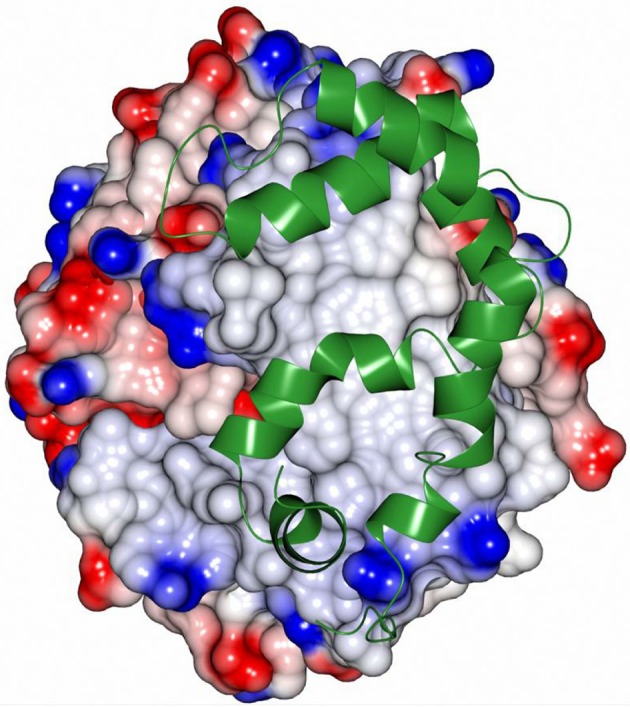
The hydrophobic interactions at the dimer interface of CH65-EH.The electrostatic surface potential of one monomer of CH65-EH that has been rotated by 90° around the vertical axis from that presented in Figure 7 is shown alongside a ribbon representation of the cap domain of the adjacent monomer (green). The areas of positive charge are shown in blue, with the areas of negative charge in red and the hydrophobic surfaces are represented in light gray.

Sibe-EH is monomeric in the crystal structure and in solution which is similar to many other bacterial EHs and plant, such as potato, EHs (Mowbray et al., 2006). CH65-EH forms a dimer in solution according to its elution profile on the size exclusion chromatography column and in the crystal structure, as suggested by PISA (Krissinel and Henrick, 2007). Within the formation of the dimer of CH65-EH (shown in Figure 7) 1,090 Å^2^ or 9% of the available surface area of the monomers is buried. The dimer interface is formed by the cap domains of the two monomers as shown in Figure 7. The interactions on the dimer interface are mainly hydrophobic (Figure 8) although several H-bonds are also formed upon dimer formation.

Known multi-domain mammalian murine and human EHs (Argiriadi et al., 1999; Pilger et al., 2015) form a dimer where both domains are involved in adjacent monomer interactions. The main contact is via the formation of an inter-subunit β-sheet involving strands β1 of the EH domains of both monomers. Interestingly, this inter-subunit β-sheet is preserved in the crystal structure of a truncated single domain human EH (Thalji et al., 2013) which is reported to be a monomer. The *Agr. radiobacter* EH is reported to be a monomer, although in its crystal structure four monomers form a tetramer with point group symmetry 222 and around 10% of the accessible surface area is buried upon tetramer formation, suggesting the possibility of a stable tetramer in solution. None of the monomer contacts within the *Agr. radiobacter* EH structure are similar to the interactions within the CH65-EH dimer. The *Nostoc* sp. EH (pdb 3QYJ), *Asp. niger* EH (Reetz et al., 2009) and insect EH from the silkworm *Bombyx mori* (Zhou et al., 2014) all form dimers via interactions of the cap domain, similar to those in CH65-EH, however the relative orientation of the monomers differs between these different EH dimers.

The nucleophile Asp102 in Sibe-EH (Asp101 in CH65-EH), is complemented by His277 and Asp249 (His270 and Asp241) to form a catalytic triad. The oxyanion hole is formed by the main chain nitrogen's of Phe35 and Trp103 (Phe34 and Trp102 in CH65-EH). Phe35 is followed by a *cis*-Pro residue conserved in other known EHs. The epoxide oxygen is co-ordinated by the side chain hydroxyls of Tyr150 and Tyr209 (Tyr148 and Tyr209 in CHE65-EH) where the tyrosine residues are located on the two adjacent helices of the cap domain.

Since the catalytic triad residues and most other residues implicated in the EH catalysis are conserved between eukaryotic and bacterial EHs it appears that the overall shape of the catalytic funnel and the precise positioning of the catalytic residues are important to explain the differences in substrate specificity.

**Figure 9:**
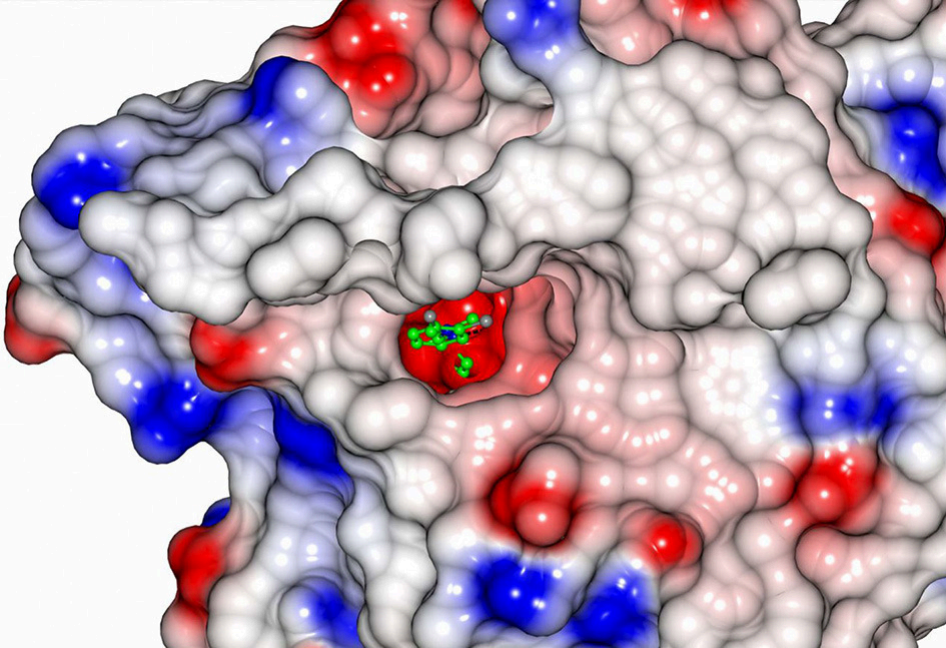
An electrostatic surface potential representation showing the entrance to the active site cavity of Sibe-EH (colors as explained in legend of Figure 8). A ligand 3-(3,4-dichlorophenyl)-1,1-dimethyl-urea that was bound to human EH (pdb code 4C4X; Pilger et al., 2015) is shown as a ball-and stick model. The position of the ligand was obtained by superimposition of the human structure onto the structure of Sibe-EH.

All EH enzymes have a deep and hydrophobic active site cavity on the interface of the two domains (Kong et al., 2014). The volumes of the active site cavities were calculated using the program CASTp (Tian et al., 2018) with a probe radius of 1.4 Å. These are 239 Å^3^ in CH65-EH and 215 Å^3^ in Sibe-EH. Although the active site cavities of CH65-EH and Sibe-EH appear to have the same regions as described for other EHs (Figure S6), their funnel entrance to the active site cavity is located in a different position in relation to other known bacterial and mammalian EHs. The *B. megaterium* EH has a large open active site entrance to allow easy access of the substrate to the catalytic residues, compared to the human EH and *Agrobacterium* EH which have smaller active site funnel entrances at the same location. In the case of CH65-EH and Sibe-EH the entrance to the active site cavity as seen in *B. megaterium* EH is totally obstructed by the adjacent monomer in the CH65-EH dimer and by a very different conformation in Sibe-EH in the region of residues 159–180. Instead, there is a separate small active site entrance funnel on the other side of protein monomer (Figure 9). The position of this funnel is approximately the same in CH65-EH and Sibe-EH, although it appears significantly more occluded in the CH65-EH enzyme. There is no access to the active site cavity in this region of the protein in the *B*. *megaterium* EH or the human EH.

### Structural basis for EH thermostability

The CH65-EH was shown to be significantly more thermostable than Sibe-EH, with no decrease of activity after incubation at temperatures of 60°C and retaining about 80% of the initial activity after thermal treatment at 70°C, while Sibe-EH was relatively stable at temperatures below 40°C and was able to retain 10% of initial activity after incubation at 60°C. This was also confirmed by CD analysis as described earlier. When analyzing the structures of the enzymes the higher thermostability of CH65-EH appears to result from a significant amount of hydrophobic interactions on the dimer interface (Figure 8) and a higher proportion of secondary structure in this enzyme (56.8%) as calculated by PROMOTIF (Hutchinson and Thornton, 1996). Whereas, the Sibe-EH has a lower proportion of secondary structure (53.3%) and is only a monomer. In comparison, the structure of a previously studied human EH hydrolase domain (PDB 4C4X) has a lower proportion of secondary structure of 49%. There are many general structural features that have been identified which are thought to contribute to protein thermostability which varies amongst different organisms (Littlechild et al., 2007; Littlechild, 2015). Examples of these features include using hydrophobic interactions at oligomeric interfaces which are often observed in thermostable proteins with temperature optima up to approximately 75°C such as the *Sulfolobus solfataricus* serine transaminase (Sayer et al., 2012) and *Thermococcus litoralis* pyrrolidone carboxyl peptidase (Singleton et al., 1999).

### Structural basis for EH substrate specificity and stereospecificity

The substrate range of the enzyme is determined by two structural components. The compound is an unlikely substrate if it cannot reach the catalytic site due to substrate channel limitations. Provided the compounds are able to get into the active site the activity depends on the potential substrate positioning in relation to the catalytic residues. This positioning is defined by the residues lining the active site cavity.

The modeling studies that have been carried out as part of this study have shown that compounds (**1**)-(**6**) can easily reach the active site cavity of the Sibe-EH and CH65-EH regardless of the different position and size of the active site funnel in these enzymes. This suggests that the enzyme reactivity towards these epoxides is defined by the precise substrate positioning in the active site. None of the substrates could be docked in the catalytic position (epoxide oxygen coordinated by hydroxyls of the catalytic tyrosines and the scissile epoxide bond co-linear with the line of attack of the catalytic aspartate oxygen) even when docking calculations were performed using both flexible side chains and flexible substrates. The results of the modeling suggest that the space between catalytic aspartate and the tyrosine residues is too restrictive to allow the epoxides (**1**)-(**6**) to bind since the movement of the side chains of these residues is limited. The tyrosine residues are held in position by neighboring hydrophobic residues and the side chain of the catalytic aspartic acid by interaction with the oxyanion hole. However, since the catalytic triad residues and catalytic tyrosine residues come together from different domains a relatively small domain movement (in the range of 0.7–1.2 Å) would allow the epoxide to move into the position required for catalysis. The tyrosines 148 and 209 (CH65-EH numbering) are located on two different intersecting helices which would move as a rigid body during domain movement.

The importance of the active site cavity residues for the EH reaction has been demonstrated by van Loo et al. (2009), where a change in substrate preference and stereospecificity of *Agr. radiobacter* EH was achieved by mutation of a single residue (Phe108, which follows the catalytic Asp107).

The first stage of the EH catalytic reaction is an attack by the OD1 of the active site Asp on one of the substrate epoxide carbons. The line of attack should be co-linear with the direction of the epoxide CO bond. The substrate epoxide oxygen binds to the side chain hydroxyls of Tyr150 and Tyr209 in Sibe-EH, and its likely position is marked by a conserved water molecule interacting with these residues. The orientation and position of the catalytic Asp side chain is fixed due to its binding in the oxyanion hole. When the structures of Sibe-EH and CH65-EH are superimposed the positions of OD1 of catalytic Asp102 (Asp101) and OH of Tyr209 (Tyr209) match quite well (within 0.5 Å), however the positions of OH of Tyr150 of Sibe-EH and Tyr148 of CH65-EH differ by 1.1Å which displaces the epoxide oxygen site by 0.8 Å.

**Figure 10:**
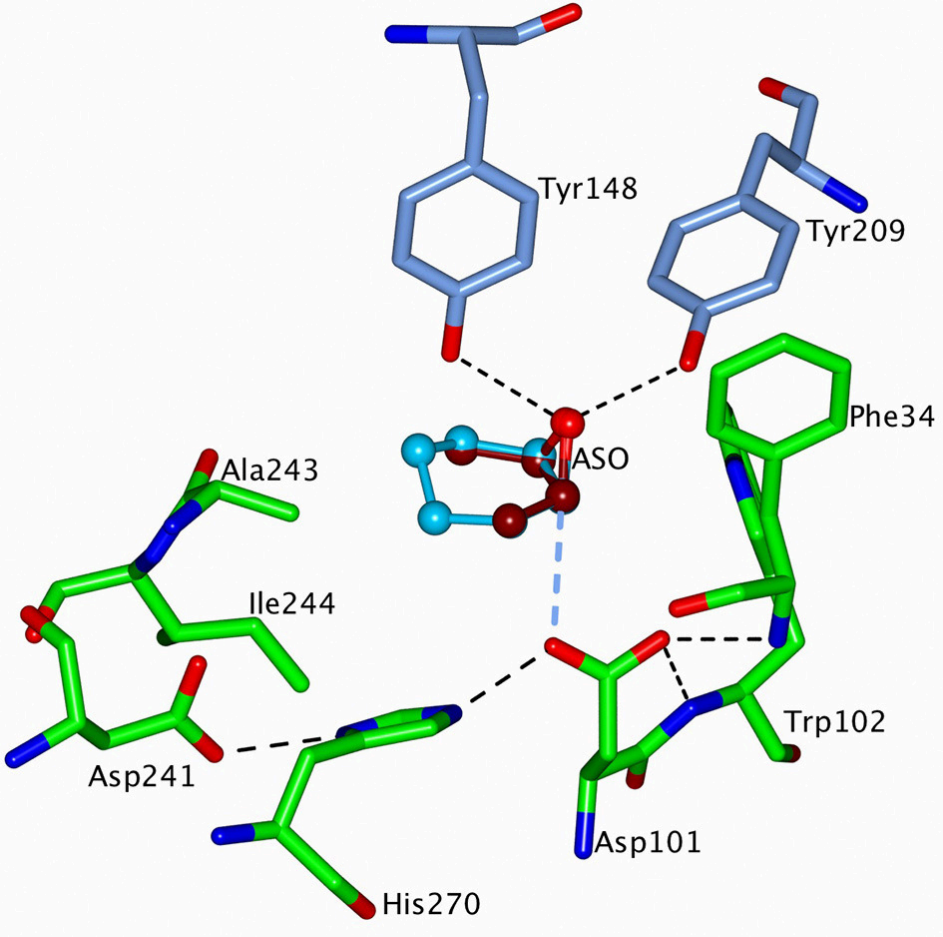
A representation of the modeled structure of CH65-EH with the putative substrates, *cis*-2,3-epoxybutane (ASO) **(3)** and cyclohexene oxide **(2)** positioned into the active site of the enzyme. ASO is shown in dark red carbons and the cyclohexene oxide in sky blue carbons. The cap domain of CH65-EH has been rotated around the line connecting the Cα atoms of residues 128 and 224 to reach a 1.2 Å displacement of OH atoms of Tyr148 and Tyr209 away from the catalytic domain. The active site residues are shown as cylinders with carbon atoms of the cap domain residues in blue and of the catalytic domain residues in green. H-bonds within the catalytic triad, in the oxyanion hole and between the epoxide oxygen and OH atoms of the two tyrosine residues, are shown as dashed black lines. The larger cyclohexene oxide is able to interact with the hydrophobic side chains of residues Ala243 and Ile244 which would lead to turnover of this substrate whereas the smaller ASO is not a substrate. Figures 6–10 were prepared using ccp4 mg (McNicholas et al., 2011).

The ligand models were positioned in the active sites of the two EHs as follows. The epoxide carbon under attack [e.g., (*S*)- or (*R*)- carbon in compound (**3**)] was aligned between OD1 of the catalytic aspartate and the epoxide oxygen. This oxygen was positioned at the location of the conserved water coordinated by the two active site tyrosine residues with the cap domain shifted (opened) by about 1.0 Å by rotation around line connecting residues 128 and 224 (CH65-EH numbering; Figure 10). The substrates were then rotated around the epoxide bond subject to attack in order to find a position where they make hydrophobic contact with the active site cavity wall and have no steric clashes. Interestingly, when ligands (**1**) and (**3**) were thus positioned and rotated in CH65-EH they appear not to make good hydrophobic contacts in any orientation. This could explain the absence of activity of CH65-EH toward these two small epoxides, since they cannot stay in the reaction position for long enough to allow catalysis to occur. However, bulkier/longer (**2**), (**4**), and (**5**) substrates make limited contacts with residues in the active site cavity, such as with residue Ile244, and are consequently turned over. The rotation of compounds (**1**) and (**3**) into the active site of Sibe-EH around a somewhat different axis allowed at least one favorable orientation to be found where the ligand interacts with Asp251 and Leu252. For both (**1**) and (**3**) this interaction exists when the (*S*)-carbon is attacked. This suggests that a hydrophobic interaction between the protein and the substrate slows down the substrate displacement and favors the reaction. The high *ee* observed for the *meso*-epoxide (**3**) in the Sibe-EH reaction can be explained by the formation of favorable protein-ligand hydrophobic interactions when the ligand is attacked at the (*S*)-carbon to release the (*R, R*) product. These interactions do not occur when the attack is at the (*R*)-carbon.

## Conclusions

Two novel EH enzymes of the α/β fold class have been identified from metagenomic samples from diverse locations in Russia and China. These two new thermostable EHs have been cloned and over-expressed in *E. coli* and have been characterized both biochemically and structurally. Both enzymes are thermostable with the CH65-EH enzyme retaining activity at 70°C.

The EHs were active toward a broad range of substrates including racemic (4*R*)-limonene-1,2-epoxide which is associated with a different class of epoxide hydrolases (LEHs) which have a different structure and mechanism and have been named by their ability to use the limonene epoxide as a substrate.

The Sibe-EH is a monomer whereas the CH65-EH enzyme forms a dimer by the interaction of its two cap domains as described for other dimeric EHs. However, the monomer orientation that makes up the dimer of CH65-EH is different from related enzymes. The funnel that forms the entrance to the active site cavity is located in a different position in CH65-EH and Sibe-EH in relation to other known bacterial and mammalian EHs.

The results obtained from this study also pose interesting questions about the evolution of the EH enzymes and their varied substrate specificities which has an important impact for their potential use as commercial biocatalysts. The high thermal stability of the CH65-EH enzyme which ranks as the most thermostable natural EH enzyme described to date, makes it an important addition to the industrial bio-catalytic “tool box.”

The detailed structural analysis of these two novel EH enzymes has allowed a greater understanding of their stability and substrate specificity. It has also demonstrated the importance of the positioning of the substrate within the active site of the enzymes to allow the catalytic turnover.

The discovery of new novel enzymes using metagenomic DNA to access the biodiversity offered by “Nature” during the HotZyme project has been clearly demonstrated within this study. This approach allows access to the large resource of enzymes encoded within the DNA of micro-organisms which are currently not able to be cultured in the laboratory.

## Accession numbers

Nucleotide sequence data are available in the GenBank databases under the accession numbers KX505385 for Sibe-EH and KX505386 for CH65-EH. The atomic coordinates and structure factors of the crystal structures have been deposited in the Protein Data Bank with the codes 5NG7 for Sibe-EH and 5NFQ for CH65-EH.

## Author contributions

EF performed the discovery, cloning and expression of the novel enzymes, while CS, SD, EG, and CM performed the protein purification. CS and SD carried out the crystallization and structure determination studies with MI carrying out data collection and analysis and structural refinement. VS carried out the molecular modeling studies with MI to rationalize the substrate specificity. EG and CM performed the biochemical characterization of functional properties and the substrate specificity studies. JL and DM coordinated the work and wrote the manuscript with input from all authors.

### Conflict of interest statement

The authors declare that the research was conducted in the absence of any commercial or financial relationships that could be construed as a potential conflict of interest.

## Abbreviations

CH65-EH: epoxide hydrolase from the metagenomic DNA from the Chinese sample
*ee*: enantiomeric excess
EH: epoxide hydrolase
LEH, limonene-1: 2-epoxide hydrolase
Sibe-EH: epoxide hydrolase from the metagenomic DNA from the Tomsk sample.
