# Supplementary material for: New Thermophilic α/β Class Epoxide Hydrolases Found in Metagenomes From Hot Environments
Source: Front Bioeng Biotechnol. 2018 Oct 16;6:144. doi: 10.3389/fbioe.2018.00144 (PMC6198070; doi:10.3389/fbioe.2018.00144)
Supplement: Supplementary file 1 [file Data_Sheet_1.PDF]

Supplementary Material

New thermophilic α/β class epoxide hydrolases found in metagenomes from hot environments

Erica Elisa Ferrandi, Christopher Sayer, Simone Antonio De Rose, Elisa Guazzelli, Carlotta Marchesi, Vahid Saneei, Michail N. Isupov, Jennifer A. Littlechild*, Daniela Monti*

*** Correspondence:** Jennifer A. Littlechild: J.A.Littlechild@exeter.ac.uk; Daniela Monti: daniela.monti@icrm.cnr.it

Doc S1. Rare codons in EH homologue gene sequences

Rare codons in the EH homologue gene sequences are shown underlined and coloured according to red codons code for Arg, green codons code for Leu, orange codons code for Pro and blue codons code for Ile.

Sibe-EH:

*atg acc ttc gaa ctc aag cgc gtg gcg ctg **CCC** aac ggt atc cat ctc gac gtg gtg gac gaa ggc **CCC** acc gat gcg ccg gtg ctg atc ttc ctc cac ggc ttt cct gaa agc cac cgc acc tgg cgc cac cag atc cgc cat ttc tcc gac cgc ttc cgc tgc att gcc **CCC** gac cag cgc ggc tat cgc ggc tcg tcc aag ccg cag gag gtc gcc gcc tat acg **CCC** gac aag ctg atc ggc gac atc ttc **CTA** ctg gcc gac acg ctg ggg atc ggc agc ttc acc atc gtc ggg cac gac tgg ggc ggc gcg att gcc tgg ggc gtg gcg ctg ggc ggg cag cac ctg cgg gtc gag cgt gcg atc atc gcc aat gcc ccg cac ccg gcg atc ttc cag aag ctg ctc tac acc cac **CCC** gtc cag cgc gag gcg agc cag tat atc cgc ggc ttc cgc gat ccg gcc aac gat gcg ctg gtg aag gaa cac ggg ctc acc gga ctg ctg atg aag gag gtc aag tgg gat cgc **CCC** agc gcg atg gag **CCC** gag gaa cgc gac cag ctg ctg cgc gac tgg cag aac cac gat gcg gcc ttc ggg atg ctc aac tat tac cgc gcg agc **CCC** atc gac gtg ccg acg atg gat gcg **CCC** ttc aaa gtg **CCC** gcc ggc tat acc ccg ccg cag ctt **CCC** **AGG** ctg acc atc **CCC** acc ctg gtg atc tgg gcg ctc gac gat ctc gcc ctt **CCC** cct gag aac ctc gaa ggg ctg gag gag atc atc gac ccg ctc acc atc gtg cgc gtg **CCC** gat tgc ggg cat ttc gtg ccg tgg gaa gcg ccg gac gcg gtc aat gcg gcg atg gag gaa ttc ctc gcg ggc taa*

CH65-EH:

*atg aac gaa atg tta aaa cac gaa tat gtc aaa gtc aat gga atc aaa atg cat tat gta acg cag gga aaa gga aaa **CTA** ttg ctt ttg tta cat ggt ttt ccg gac ttt tgg tat gtt tgg cgt ttt caa att cca gca ctg gct aag cat ttt cgt gtc gtt gct cca gac tta **AGG** ggt tac aac gag acg gac aag ccg gaa ggt gtc gag aat tat **CGA** ttg gat ctt ctc gca aaa gac att tta gga ctc att aaa gct ctc ggc gaa gag cat gca gtg gtt gtt ggg cat gac tgg ggt ggg atc atc agc tgg act ctc acc gct ttt aat ccg caa gca gtt gag aag ctg gta att tta aat gcg cct cat **CCC** aaa gcc tat atg act **CGA** act aaa aat tcg **CTA** **AGG** cag ttg caa aaa agc tgg tat gtc ttc ttt ttc cag gtg gcg aac att cca gag aag att ttg agt **CGA** aat gag ttt gct ttt ctt aaa aac atg ctc att caa tct ttt gtc **AGA** **AGA** gat ctt ctt aca gaa gag gac tta **AGG** atc tac gtt gat gct tgg tcc aaa tcg gga gct tta act tct gca ttg aat tac tat **AGA** gcg aat **CTA** aat cca gac **ATA** att ttt tca gag aaa act gta gtc ttt cca aag atc aaa gtg **CCC** acc ttg gtg att tgg gga gaa aaa gat gtg gcg atc tca aaa gat tta **ATA** gtg aac atg gag gac ttc att gaa gct cca tac tct **ATA** aag tat ttc ccg gaa tgt ggg cac tgg gtc cag ctt gaa gag cct gag ctt gtc **AGA** aaa cac **ATA** gag gag ttt **ATA** ttg aaa tcg gat att taa*

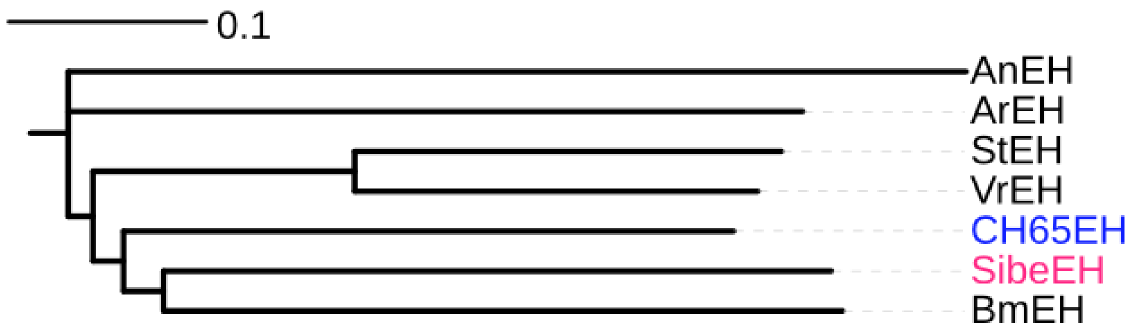

Figure S1: Phylogenetic analysis of Sibe-EH and CH65-EH with known selected EHs

The phylogenetic tree was created using the Clustal Omega webserver (http://www.ebi.ac.uk/Tools/msa/clustalo/) and visualized using the iTOL webserver (http://itol2.embl.de/). AnEH=*Aspergillus niger* EH, GenBank CAB59812.1, PDB 1QO7_A; ArEH= *Agrobacterium radiobacter* EH, PDB: 1EHY_A; StEH=Solanum tuberosum EH, PDB: 2CJP_A; VrEH=*Vigna radiata* EH, GenBank ADP68585.1, PDB 5XMD_A; BmEH=*Bacillus megaterium* EH, GenBank ADV36302.1 PDB 4O08_A.

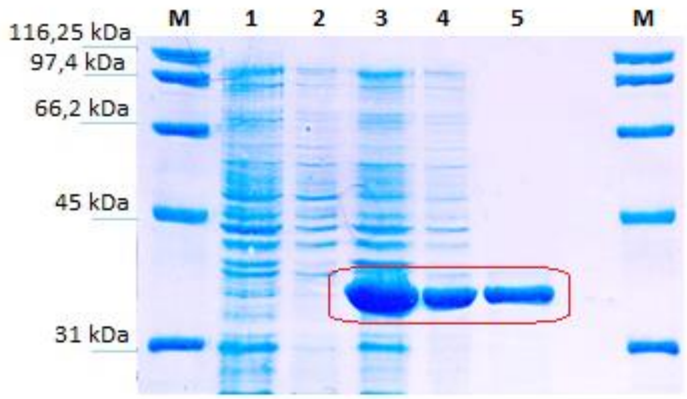

Figure S2: Recombinant expression in *E. coli* BL21 RIPL and purification of Sibe-EH

M, MW markers; 1, total cell extract of un-induced cells; 2, supernatant of un-induced cells; 3, total cell extract of induced cells; 4, supernatant of induced cells; 5, Sibe-EH after Ni-NTA affinity chromatography

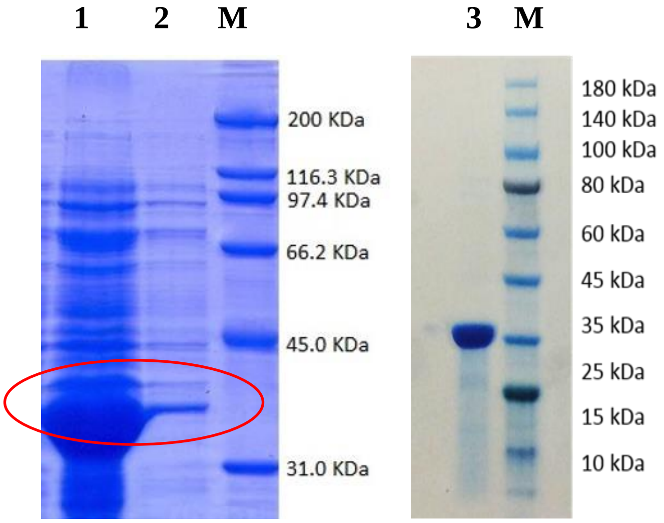

Figure S3: Recombinant expression in *E. coli* BL21(DE3) of the codon-optimized CH65-*eh* gene and CH65-EH purification

M, MW markers; 1, total cell extract of induced cells; 2, supernatant of induced cells; 3, purified CH65-EH after Ni-NTA affinity and size exclusion chromatography

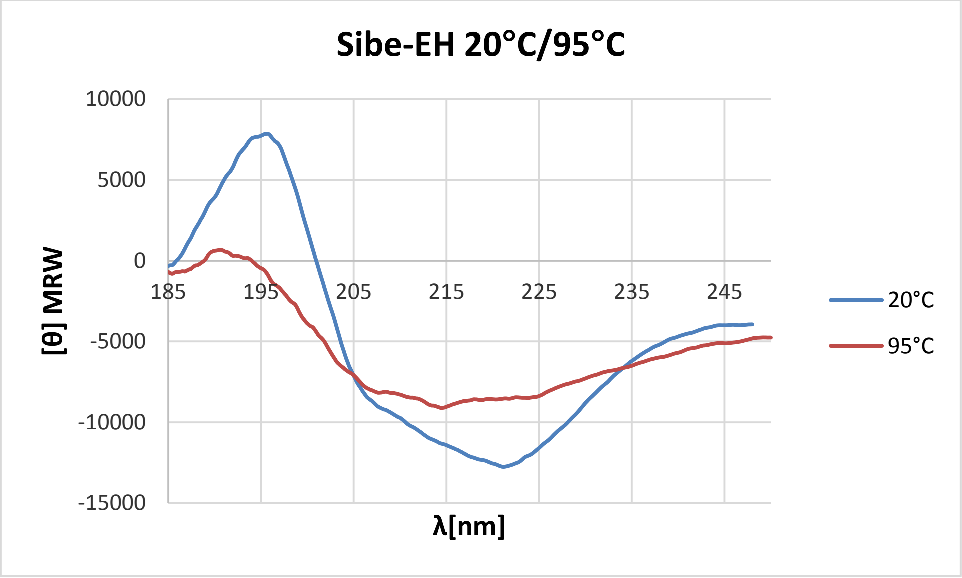

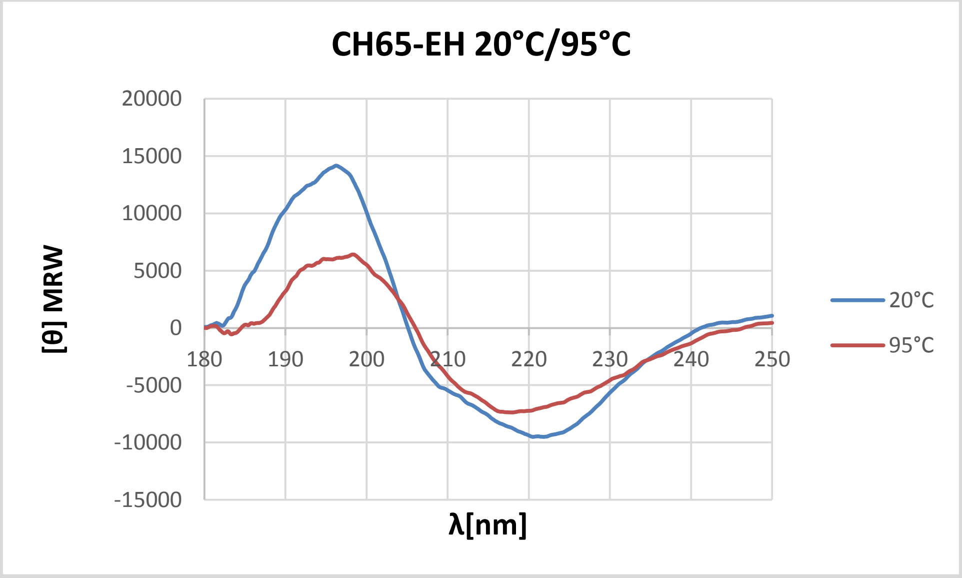

Figure S4: CD analysis of EHs

Circular dichroism analysis of Sibe-EH and CH65-EH carried out at 20°C or 95°C

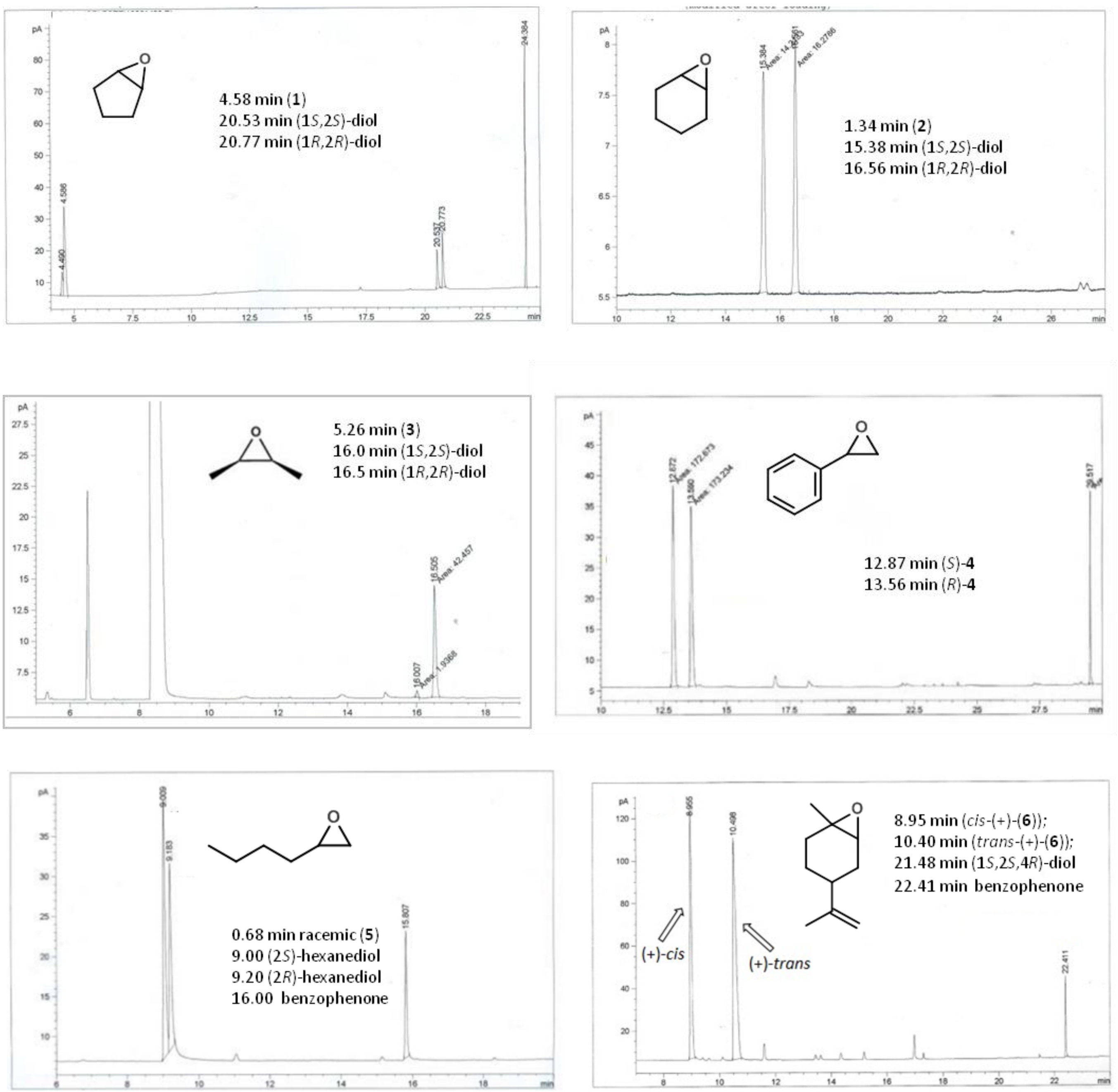

Figure S5: GC chromatograms of substrates 1-6 and corresponding diol products

Table S1. Primers used in this study. Primers F1 and R1 were used for the Sibe-*eh* gene region amplification. In particular, primer F1 shows a sequence complementary to the flanking region upstream Sibe-*eh* gene, while primer R1 shows a sequence reverse complementary to the flanking region downstream the Sibe-*eh* gene. Primers F2 and R2 were used for CH65-*eh* gene amplification. The primers are complementary to the 3'end and 5' end region of the CH65-*eh* gene, respectively. Primers F3/R3 and F4/R4 were used for the amplification of Sibe-*eh* and CH65-*eh* respectively, and include a 18 nucleotide overlap with the ends of the pRham vector for the subsequent cloning into the pRham plasmid. Primers F5/R5 were used for the amplification of the optimized CH65-*eh* gene. F5/R5 are complementary to the 3'end and 5' end region of the CH65-*eh* optimized gene and include 18 nucleotide overlap with the ends of the pETite vector for the subsequent cloning into the pETite plasmid. Primers F6/R6, F7/R7-8 and F8/R7-8 were used for the sequencing on both strands of the insert present in the pJet, pRham and pETite vectors respectively.

	Primers	**Sequence**
**F1**	*Sibe*EH-upstream	5' ACATGGTCGCCAATGGCTACAT 3'
**R1**	*Sibe*-EH-downstr.	5' GGAAGTGCAGCTCGCCTGG 3'
**F2**	*CH65*-EH-FW	5' ATGAACGAAATGTTAAAACACGAA 3'
**R2**	*CH65*-EH-RV	5' AATATCCGATTTCAATATAAACTC 3'
**F3**	*Sibe*-EH-pRhamFW	5' GAAGGAGATATACATATGACCTTCGAACTCAAGCGCGTG 3'
**R3**	*Sibe*-EH-pRhamRV	5' GTGATGGTGGTGATGATGGCCCGCGAGGAATCCCTCCAT 3'
**F4**	*CH65*-EH- pRhamFW	5' GAAGGAGATATACATATGAACGAAATGTTAAAACACGAA 3'
**R4**	*CH65*-EH pRhamRV	5' GTGATGGTGGTGATGATGAATATCCGATTTCAATATAAACTC 3'
**F5**	*CH65*-EHopt-pETiteFW	5' GAAGGAGATATACATATGAACGAAATGTTGAAACATGAA 3'
**R5**	*CH65*-EHopt-pETiteRV	5' GTGATGGTGGTGATGATGGATGTCGGACTTTAG 3'
**F6**	pJET1.2 Forward	5' CGACTCACTATAGGGAGAGCGGC 3'
**R6**	pJET1.2 Reverse	5' AAGAACATCGATTTTCCATGGCAG 3'
**F7**	pRham Forward Primer	5' GCTTTTTAGACTGGTCGTAGGGAG 3'
**F8**	pETite Forward Primer	5' TAATACGACTCACTATAGGG 3'
**R7-8**	pETite Reverse Primer	5' CTCAAGACCCGTTTAGAGGC 3'

A

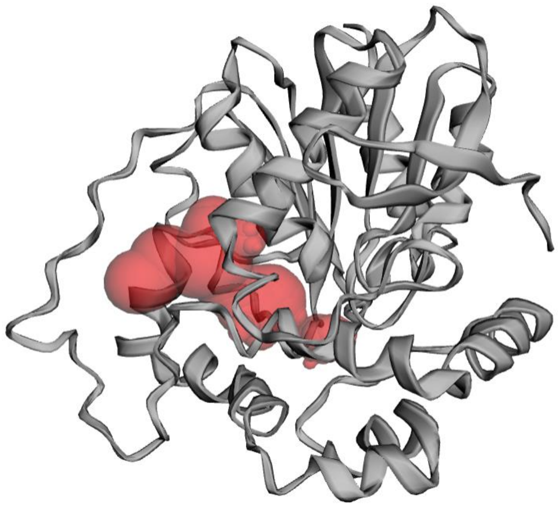

B

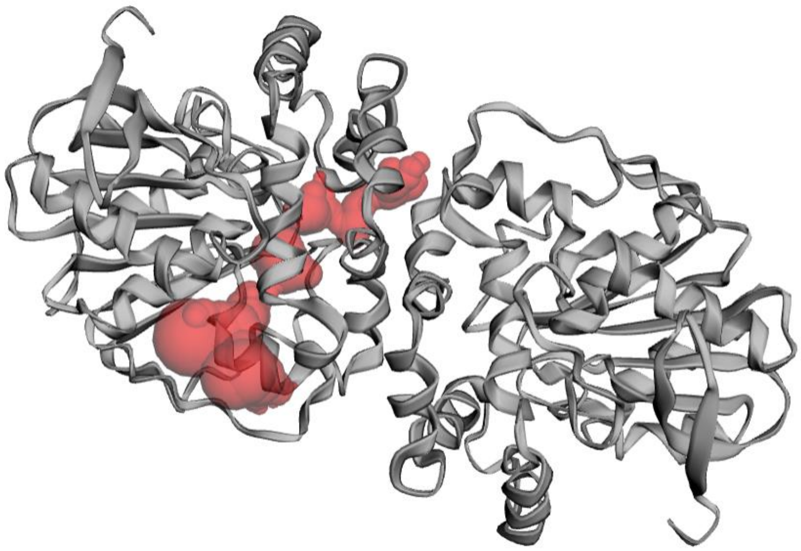

Figure S6: The active site cavities in A) Sibe-EH and B) CH65-EH shown as red space filling models. Constructed using the program CASTp (Tian et al., 2018).
